# The EIN3 transcription factor *GmEIL1* improves soybean resistance to *Phytophthora sojae*

**DOI:** 10.1111/mpp.13452

**Published:** 2024-04-15

**Authors:** Xi Chen, Yan Sun, Yu Yang, Yuxin Zhao, Chuanzhong Zhang, Xin Fang, Hong Gao, Ming Zhao, Shengfu He, Bo Song, Shanshan Liu, Junjiang Wu, Pengfei Xu, Shuzhen Zhang

**Affiliations:** ^1^ Key Laboratory of Soybean Biology of Chinese Education Ministry Soybean Research Institute of Northeast Agricultural University Harbin China; ^2^ Crop Stress Molecular Biology Laboratory Heilongjiang Bayi Agricultural University Daqing China; ^3^ Key Laboratory of Soybean Cultivation of Ministry of Agriculture Soybean Research Institute of Heilongjiang Academy of Agricultural Sciences Harbin China; ^4^ Plant Science Department, School of Agriculture and Biology Shanghai JiaoTong University Shanghai China

**Keywords:** EIN3‐binding sequence, ethylene, *GmEIL1*, Phytophthora root rot of soybean

## Abstract

Phytophthora root and stem rot of soybean (*Glycine max*), caused by the oomycete *Phytophthora sojae*, is an extremely destructive disease worldwide. In this study, we identified *GmEIL1*, which encodes an ethylene‐insensitive3 (EIN3) transcription factor. *GmEIL1* was significantly induced following *P. sojae* infection of soybean plants. Compared to wild‐type soybean plants, transgenic soybean plants overexpressing *GmEIL1* showed enhanced resistance to *P. sojae* and *GmEIL1*‐silenced RNA‐interference lines showed more severe symptoms when infected with *P. sojae*. We screened for target genes of GmEIL1 and confirmed that GmEIL1 bound directly to the *GmERF113* promoter and regulated *GmERF113* expression. Moreover, GmEIL1 positively regulated the expression of the pathogenesis‐related gene *GmPR1*. The GmEIL1‐regulated defence response to *P. sojae* involved both ethylene biosynthesis and the ethylene signalling pathway. These findings suggest that the GmEIL1‐*GmERF113* module plays an important role in *P. sojae* resistance via the ethylene signalling pathway.

## Graphical abstract

GmEIL1 promotes GmERF113 expression and the accumulation of ethylene to enhance the resistance to *Phytophthora sojae* in soybean, which provides insights into the mechanism underlying defence responses.

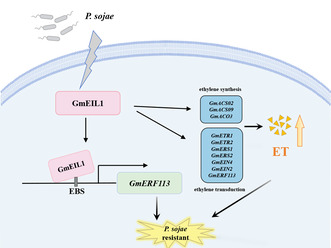

## 1 INTRODUCTION

Phytophthora root and stem rot of soybean (*Glycine max*), caused by *Phytophthora sojae*, is a destructive disease that poses a serious threat to soybean production worldwide (Schmitthenner, 1972; Tyler, 2007). Breeding for dominant resistance to *P. sojae* is an efficient and environmentally friendly way to protect soybean from *P. sojae* infection, minimizing the need for fungicide application. However, although breeders have used *Resistance to P. sojae* (*Rps*) genes to control the disease (Sugimoto et al., 2012), emerging races of this highly variable pathogen can bypass genetic resistance. Therefore, to better understand the mechanism of soybean resistance to *P. sojae* and to develop new strategies for controlling Phytophthora root and stem rot, it is imperative to study the signal transduction mechanisms and genes responsible for resistance to this destructive pathogen.

Ethylene is a gaseous hydrocarbon phytohormone that modulates plant growth and development, fruit ripening and abscission, and stress responses via a well‐studied signalling pathway (Abiri et al., 2017; Ju & Chang, 2015; Yang et al., 2017; Zhu et al., 2023). Ethylene signalling also plays an important role in plant disease resistance (Dong et al., 2020; Iwai et al., 2006; Wang et al., 2013; Yang et al., 2017). Many studies have shown that ethylene biosynthesis and downstream ethylene signal transmission are involved in host–pathogen interactions (Cabrera et al., 2014; Helliwell et al., 2013; Hossain et al., 2017; Hu et al., 2017; Nahar et al., 2012; Seo et al., 2010). For example, infection with soybean mosaic virus activated ethylene signalling in soybean seedlings, suggesting a key role in regulating the antiviral defence response (Zhang, Shang, et al., 2019). Ethylene signalling pathways can positively regulate the defence response of Chinese ginseng (*Panax notoginsen*g) roots to *Fusarium solani* (Liu et al., 2019). Following inoculation of rutabaga (*Brassica napus* subsp. *rapifera*) roots with *Plasmodiophora brassicae*, ethylene‐responsive factors were up‐regulated in the resistant cultivar Wilhelmsburger, indicating activation of ethylene‐mediated defences in the resistance response (Zhou et al., 2020). In many cases, pathogen challenge triggers enhanced ethylene production in plants (Cohn & Martin, 2005; Penninckx et al., 1998) by activating genes involved in ethylene biosynthesis (Bleecker & Kende, 2000; Wang et al., 2002).

Ethylene biosynthesis results from conversion of *S*‐adenosylmethionine (SAM) to 1‐aminocyclopropane‐1‐carboxylic acid (ACC) by ACC synthase (ACS), the rate‐limiting enzyme in ethylene biosynthesis (Kende, 1989, 1993; Yang & Hoffman, 1984). ACC is then converted to ethylene by ACC oxidase (ACO). ACS and ACO isoforms are encoded by multigene families in all plant species studied, with *Arabidopsis thaliana* having 12 *ACS* genes and five *ACO* genes (Bleecker & Kende, 2000; Johnsson & Ecker, 1998; Wang et al., 2002). The ethylene biosynthetic genes in the ACS and ACO families are often involved in plant disease resistance. For instance, *OsACS1* and *OsACS2* are induced when rice (*Oryza sativa*) plants are inoculated with the rice blast fungus *Magnaporthe oryzae*, suggesting that ethylene biosynthesis plays a role in rice resistance to this pathogen (Helliwell et al., 2016). Similarly, *OsACS2* and *OsACO7* are induced when young rice leaves are inoculated with *Magnaporthe grisea* (Iwai et al., 2006), a similar pathogen. In *Pseudomonas syringae* pv. *tomato*‐infected tomato (*Solanum lycopersicum*) plants, the bacterial effector proteins AvrPro and AvrPtoB activate expression of *LeACO1* and *LeACO2* (Cohn & Martin, 2005).

The ethylene transduction pathway is initiated when ethylene is perceived by a member of the membrane‐associated receptor family that includes ethylene response 1 (ETR1), ETR2, ethylene response sensor 1 (ERS1), ERS2, and ethylene‐insensitive 4 (EIN4) (Hall et al., 2000; Shakeel et al., 2013; Wang et al., 2017). Constitutive triple response 1 (CTR1) is a Raf‐like serine/threonine kinase that negatively regulates the ethylene signal transduction pathway (Ju & Chang, 2015), whereas EIN2, EIN3, and EIN3‐LIKE (EIL) are positive regulators of ethylene signalling (Kendrick & Chang, 2008; Qiao et al., 2012). EIN3 and EIL1 are crucial transcription factors in the ethylene signalling pathway (Alonso et al., 2003; An et al., 2010; Chao et al., 1997). They activate transcription factors such as the ethylene response factors (ERFs), thereby regulating the expression of additional downstream genes involved in the plant response to ethylene (Guo & Ecker, 2004; Kendrick & Chang, 2008; Potuschak et al., 2003).

EIN3 and EILs are sequence‐specific DNA‐binding proteins that bind to an EIN3‐binding sequence (EBS: A[T/C]G[T/A]A) in the promoters of a variety of downstream target genes that are involved in the response to ethylene (Chen et al., 2009; Konishi & Yanagisawa, 2008; Kosugi & Ohashi, 2000; Solano et al., 1998; Yamasaki et al., 2005; Zhang et al., 2011). Therefore, by activating the expression of multiple downstream genes, EIN3 and EILs regulate many physiological processes, including plant growth and development, seedling photomorphogenesis, and plant stress responses (An et al., 2012; Peng et al., 2014; Shi et al., 2012; Zhang et al., 2011; Zhong et al., 2009). For instance, *Arabidopsis* EIN3 specifically binds to the promoters of genes involved in salt tolerance, such as *ethylene and salt‐induced ERF* (*ESE1*) (Zhang et al., 2011) and *salt‐induced and EIN3/EIL1‐dependent* (*SIED1*) (Peng et al., 2014), thereby regulating their expression to increase tolerance to salt stress. In addition, EIN3 negatively regulates ethylene biosynthesis and ethylene signalling–mediated freezing tolerance in *Arabidopsis* by binding to the promoters of cold‐inducible *C‐repeat Binding Factor* (*CBF*) and type‐A *Arabidopsis response regulator* (*ARR*) genes and suppressing their expression (Shi et al., 2012).

In a previous study, we identified a strongly up‐regulated *EIN3* homologue in the highly resistant soybean cultivar Suinong 10 following infection with *P. sojae* (Xu et al., 2012). In the present study, we isolated the gene encoding this EIN3‐like transcription factor, designated *GmEIL1*, from Suinong 10 soybean plants. We then characterized *GmEIL1*, determined its tissue‐specific expression, and monitored its expression in response to *P. sojae* and ethylene. Furthermore, we generated overexpressing and silenced transgenic soybean plants to investigate the involvement of *GmEIL1* in the ethylene signalling pathway and in resistance to *P. sojae*. We found that *GmEIL1* encodes a nucleus‐localized transcription factor that responds to *P. sojae* infection by activating the downstream gene *GmERF113*, leading to improved resistance of soybean to *P. sojae*.

## 2 RESULTS

### 2.1 Expression patterns of *GmEIL1* gene in soybean and the characteristics of GmEIL1 protein

**FIGURE 1:**
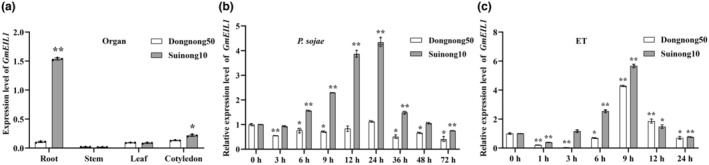
Expression patterns of *GmEIL1* in *Phytophthora sojae*‐resistant soybean cultivar Suinong 10 and *P. sojae*‐susceptible soybean cultivar Dongnong 50. (a) Spatial expression patterns of *GmEIL1* in different organs of Suinong 10 and Dongnong 50 under normal conditions. Transcript levels were normalized to *GmEF1b* levels using the 2^−Δ*C*t^ method. (b) Relative expression of *GmEIL1* in Suinong 10 and Dongnong 50 at 0 to 72 h following *P. sojae* infection (hpi). Relative expression levels of *GmEIL1* were compared to those in negative control plants (plants not infected by *P. sojae*) at the same time point, and those at each time point were normalized to the level 0 hpi. (c) Relative expression of *GmEIL1* in Suinong 10 and Dongnong 50 in response to exogenous ethylene (ET) treatment for 0, 1, 3, 6, 9, 12, and 24 h. Fourteen‐day‐old plants were used for treatments and analyses. Relative expression levels of *GmEIL1* were compared with those in negative control plants (plants treated with sterile water) at the same time point and those at each time point were compared to the corresponding 0 hpi. *GmEF1b* was used as an internal control to normalize all data. The statistical analyses in (a–c) were performed on three biological replicates, each with three technical replicates. Data were analysed using Student's *t* test (*p < 0.05, **p < 0.01). Error bars indicate the standard errors of the means.

To evaluate the expression of *GmEIL1*, we determined the transcript levels of *GmEIL1* by reverse transcription‐quantitative PCR (RT‐qPCR) in roots, stems, leaves, and cotyledons of the resistant soybean cultivar Suinong 10 and the susceptible soybean cultivar Dongnong 50. Expression levels of *GmEIL1* were much higher in roots and cotyledons of Suinong 10 than in Dongnong 50, and low in stems and leaves of both soybean cultivars (Figure 1a). We further explored the expression patterns of *GmEIL1* in soybean plants inoculated with *P. sojae* and compared expression levels at each time point to the corresponding level at 0 hours post‐inoculation (hpi). By 6 hpi, *GmEIL1* expression was significantly induced by *P. sojae* in the leaves of Suinong 10, reaching the highest value at 24 hpi, after which it dropped sharply (Figure 1b). In contrast, there was no significant increase in *GmEIL1* transcript levels in the leaves of Dongnong 50 after treatment with *P. sojae*, demonstrating that the resistant and susceptible cultivars showed different *GmEIL1* expression patterns (Figure 1b).

Following ethylene treatment, the relative expression of *GmEIL1* decreased initially (at 1 h post‐treatment), then significantly increased, reaching a maximum level at 9 h in both Suinong 10 and Dongnong 50 (Figure 1c). These results suggested that *GmEIL1* is involved in ethylene signalling pathways in response to *P. sojae*.

We produced the full‐length cDNA sequence of *GmEIL1* (GenBank accession no. XM_003545435) from total RNA of Suinong 10 soybean leaves by reverse transcription‐PCR (RT‐PCR). Sequence analysis of *GmEIL1* revealed a 2576‐bp cDNA containing a 1833‐bp open reading frame encoding a polypeptide of 610 amino acid residues, with a predicted molecular mass of 69 kDa. The predicted structure of GmEIL1 included a conserved 125‐residue EIN3 DNA‐binding domain (Figure S1a).

Alignment and phylogenetic analysis of GmEIL1 and related protein sequences revealed that it belongs to the EIN3/EILs family, and that GmEIL1 is closely related to soybean GmEIL1b, GmEIL1c, and GmEIL1d, as well as to mung bean (*Vigna radiata*) VrEIL1 and VrEIL2, *Arabidopsis* AtEIL1 and AtEIN3, grapevine (*Vitis vinifera*) VvEIN3, and melon (*Cucumis melo*) CmEIN3 (Figure S1b). Analysis of the conserved EIN3 domain of 125 amino acids showed that it shares 60%–96% amino acid identity with nine of the family members (Figure S1a). The three‐dimensional structures of GmEIL1 and AtEIL1 were predicted using InterPro AlphaFold (https://www.ebi.ac.uk/interpro/), and the putative EIN3 domain is labelled in the structures (Figure S1c). Based on these data, GmEIL1 can be classified as a member of the EIN3/EIL family.

### 2.2 GmEIL1 is localized in the nucleus

**FIGURE 2:**
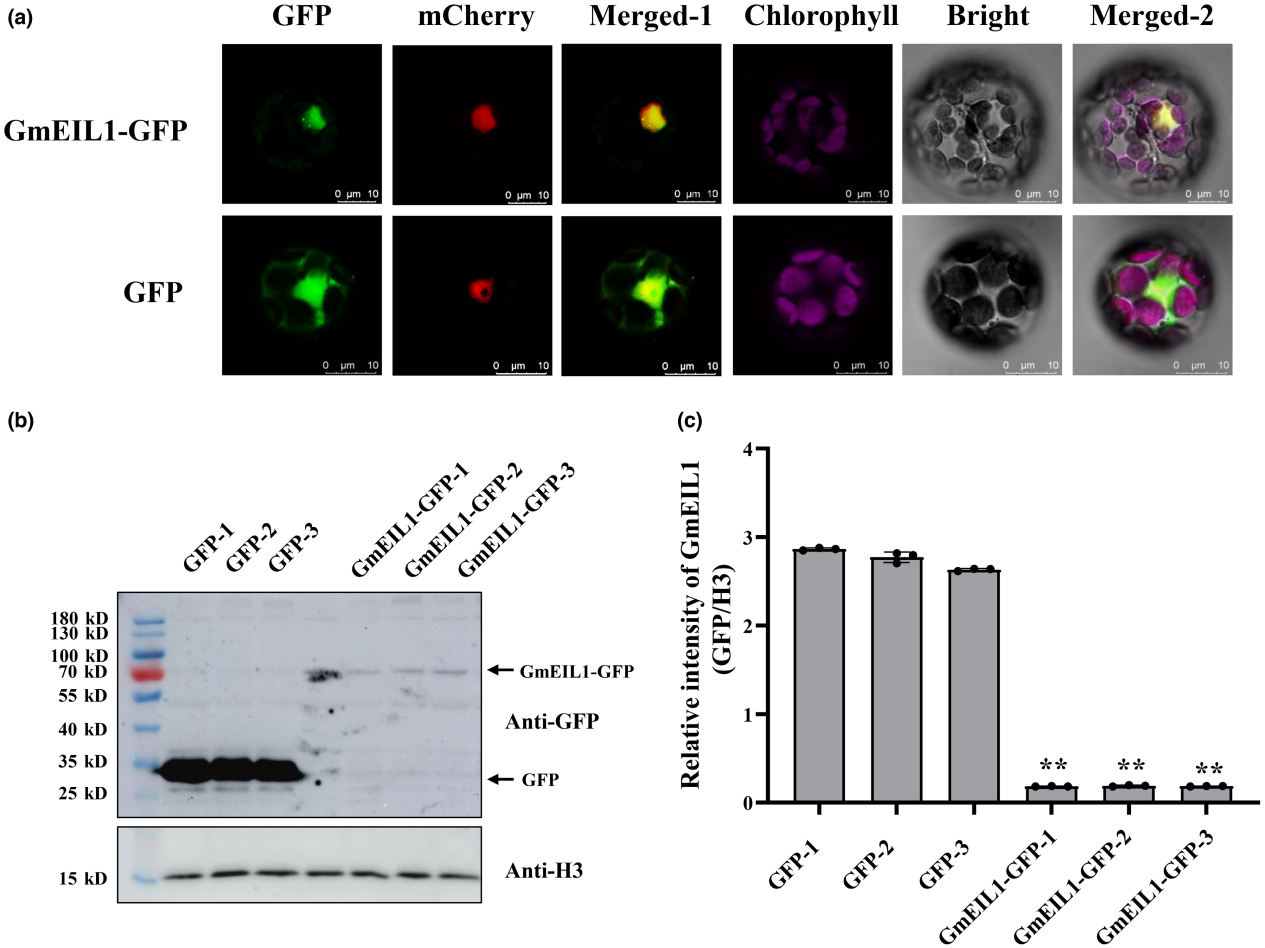
Subcellular localization of GmEIL1. (a) Fluorescence analysis of GmEIL1‐GFP and GFP localization. The GmEIL1‐GFP and H2B‐mCherry fusion plasmids (upper panels) or 35S:GFP and the H2B‐mCherry marker for the nucleus (lower panels) were cotransformed into *Arabidopsis* protoplasts using a polyethylene glycol‐mediated method. Panels show bright‐field images, GFP fluorescence (green), chlorophyll autofluorescence (purple), mCherry fluorescence (red) and merged images. Merged‐1 is a merged image of H2B‐mCherry and GFP channels and Merged‐2 is a merged image of all channels. Bars indicate 10 μm. The fluorescence images indicated that GmEIL1 localizes in the nucleus. (b) Immunoblot analysis of GmEIL1‐GFP and GFP localization in nuclei. Histone H3 was used as a nuclear marker for normalization. The arrows point to the expected proteins. (c) Quantitative measurement of immunoblot signals in panel (b). Signal intensities were quantified using ImageJ software. For relative intensity, the intensity of GmEIL1‐GFP and its corresponding nuclear marker H3 protein was first measured by ImageJ software, and then the intensity of GmEIL1‐GFP relative to H3 (GmEIL1‐GFP/H3) was analysed. Statistical significance was determined by Student's *t* test and is indicated as **p < 0.01. Error bars show the standard deviations of the means.

To examine the subcellular localization of GmEIL1, we monitored the accumulation of a GmEIL1‐GFP (green fluorescent protein) fusion protein in *Arabidopsis* protoplasts. The GFP signal was observed throughout protoplast cells harbouring a construct constitutively expressing GFP driven by the cauliflower mosaic virus 35S promoter (35S:GFP), whereas the GFP signal was strongly concentrated in the nucleus of cells transformed with a construct expressing the GmEIL1‐GFP fusion protein (Figures 2a and S2). We analysed the nuclear accumulation of GmEIL1‐GFP and GFP by immunoblotting using anti‐GFP antibodies and identified GmEIL1‐GFP at approximately 82 kDa and GFP at approximately 27 kDa (Figure 2b). The immunoblotting results were quantified in grey scale using ImageJ software to calculate the ratio of GFP or GmEIL1‐GFP relative to the nuclear marker Histone 3 (H3) (Figure 2c), and overall, these results indicated that GmEIL1 is a nucleus‐localized protein.

### 2.3 GmEIL1 enhances resistance to *P. sojae* in transgenic soybean plants

**FIGURE 3:**
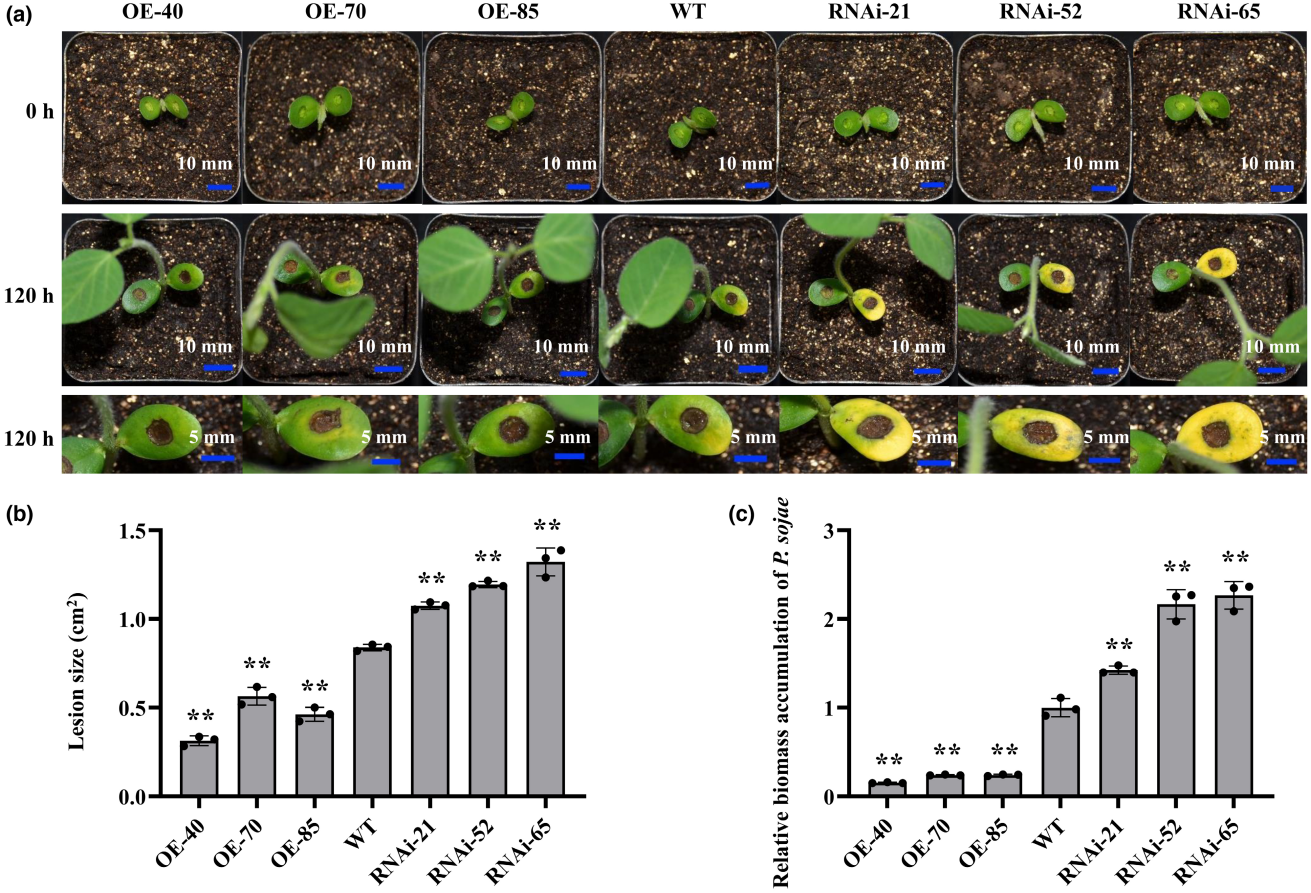
*GmEIL1* enhances resistance to *Phytophthora sojae* in transgenic soybean cotyledons. (a) Disease symptoms in cotyledons of *GmEIL1‐*OE, wild‐type (WT, Dongnong 50) and *GmEIL1*‐RNAi plants before (0 h) and 120 h after inoculation with *P. sojae*. (b) Lesion size was measured from photographs of cotyledons of plants at 120 h post‐inoculation and represents the mean lesion area for each independent soybean line (*n* = 3) compared with the mean lesion area for WT soybean plants. (c) Quantitative PCR analysis of the relative biomass of *P. sojae* in *GmEIL1* transgenic and WT soybean plants based on *P. sojae TEF1* transcript levels. The relative expression levels of *TEF1* were measured at 120 h after *P. sojae* infection. *GmEF1b* was used as the internal control to normalize all data. The statistical analyses of (b) and (c) were performed on three biological replicates, each with three technical replicates. Statistical significance was analysed using Student's *t* test (**p < 0.01). Error bars indicate the standard errors of the means.

**FIGURE 4:**
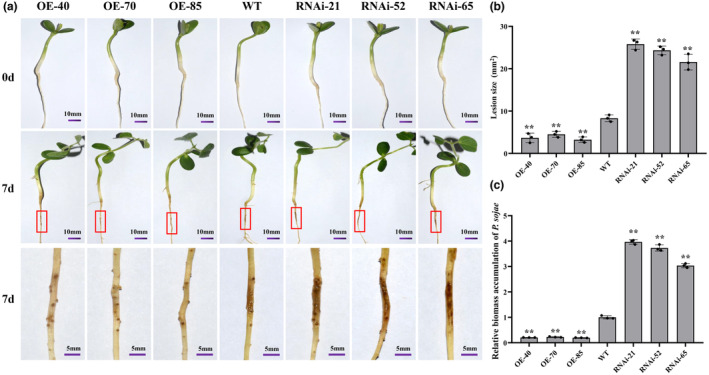
Analysis of resistance of *GmEIL1* transgenic soybean plants to *Phytophthora sojae*. (a) Disease symptoms in roots of *GmEIL1*‐OE, wild‐type (WT, Dongnong 50), and *GmEIL1*‐RNAi plants before (day 0) and 7 days after inoculation with *P. sojae*. (b) Lesion size was measured from photographs of roots of *GmEIL1*‐OE, *GmEIL1*‐RNAi, and WT plants before (day 0) and 7 days after inoculation. Bars represent the mean lesion areas for each independent soybean line (*n* = 3). (c) Reverse transcription‐quantitative PCR analysis of *TEF1* expression in *GmEIL1* transgenic and WT soybean plants based on *P. sojae TEF1* transcript levels. The relative expression levels of *TEF1* were measured and compared 7 days after *P. sojae* infection. *GmEF1b* was used as the internal control to normalize all data. Statistical analyses in (b) and (c) were performed on three biological replicates, each with three technical replicates. Data were analysed using Student's *t* test (**p < 0.01). Error bars indicate the standard errors of the means.

To investigate whether *GmEIL1* affects soybean resistance to *P. sojae*, we generated overexpressing (*GmEIL1*‐OE) and RNA interference‐silenced (*GmEIL1*‐RNAi) transgenic soybean plants. Overexpression of *GmEIL1* in three independent *GmEIL1*‐OE soybean lines (OE‐40, OE‐70, and OE‐85) was confirmed by immunoblot analysis (Figure S3a) and RT‐qPCR (Figure S3b), and silencing of *GmEIL1* in three independent RNAi soybean lines (RNAi‐21, RNAi‐52, and RNAi‐65) was confirmed by RT‐qPCR (Figure S3b). We characterized the resistance of these soybean plants to *P. sojae* in cotyledons and roots following inoculation with *P. sojae* zoospores. The cotyledons of *GmEIL1*‐OE plants showed less severe infection than did those of wild‐type (WT) Dongnong 50 plants or *GmEIL1*‐RNAi plants at 120 h after incubation with *P. sojae* (Figure 3a). In contrast, the cotyledons of *GmEIL1*‐RNAi plants exhibited water‐soaked lesions and the surrounding cotyledons turned yellow and were softer than those of the WT. The lesions on the cotyledons of the *GmEIL1*‐RNAi plants were significantly larger than the lesions on WT cotyledons but were significantly smaller than those on the *GmEIL1*‐OE plants (Figure 3b). In addition, the *P. sojae* biomass (indicated by the relative abundance of *TEF1* genomic sequence per area of infected cotyledon) was significantly lower in *GmEIL1*‐OE plants than in either WT plants or *GmEIL1*‐RNAi plants (Figure 3c). The roots of the WT soybean plants and *GmEIL1*‐RNAi plants exhibited watery and rotting lesions, whereas those of the *GmEIL1*‐OE plants remained healthy (Figure 4a). Similar to the results when infecting cotyledons, the lesion areas (relative to the WT) on the roots and the biomass of *P. sojae* 7 days after infection were significantly greater in the *GmEIL1*‐RNAi plants, but significantly less in the *GmEIL1*‐OE plants (Figure 4b,c). These results showed that *GmEIL1* could enhance soybean resistance to *P. sojae*.

In order to determine the impact of *GmEIL1* homologues on *GmEIL1* in response to *P. sojae* infestation, we initially examined the expression level of *GmEIL1b*, *GmEIL1c*, and *GmEIL1d* in Suinong 10 and Dongnong 50 plants upon *P. sojae* infection. All three homologues were induced by *P. sojae* in Suinong 10, and only *GmEIL1d* was induced by *P. sojae* in Dongnong 50, demonstrating that *P. sojae* infection affected the expression level of *GmEIL1* homologues (Figure S4).

We then analysed the transcript levels of *GmEIL1* and the three homologous genes in roots and cotyledons of three *GmEIL1*‐OE lines, three *GmEIL1*‐RNAi lines, and WT plants. The transcript levels of *GmEIL1*, but not those of *GmEIL1b* (XM_003519439), *GmEIL1c* (XM_003543111), or *GmEIL1d* (XM_003543106), were elevated significantly in roots (Figure S5) and cotyledons (Figure S6) of *GmEIL1*‐OE plants relative to WT plants. The transcript levels of *GmEIL1* and *GmEIL1b* were significantly lower in *GmEIL1*‐RNAi plants than in the WT, whereas there were no significant changes in *GmEIL1c* and *GmEIL1d* transcript levels. Because *GmEIL1b* is highly homologous to *GmEIL1*, RNAi of *GmEIL1b* by *GmEIL1* could not be avoided. The expression level of *GmEIL1b* in the *GmEIL1*‐RNAi lines was also significantly lower than in the WT. Expression of the other two homologous genes (*GmEIL1c* and *GmEIL1d*) was not altered in *GmEIL1*‐RNAi plants. In summary, the above results indicated that *GmEIL1* enhances soybean resistance to *P. sojae*.

### 2.4 GmEIL1 regulates *GmERF113* expression by binding to the *GmERF113* promoter

**FIGURE 5:**
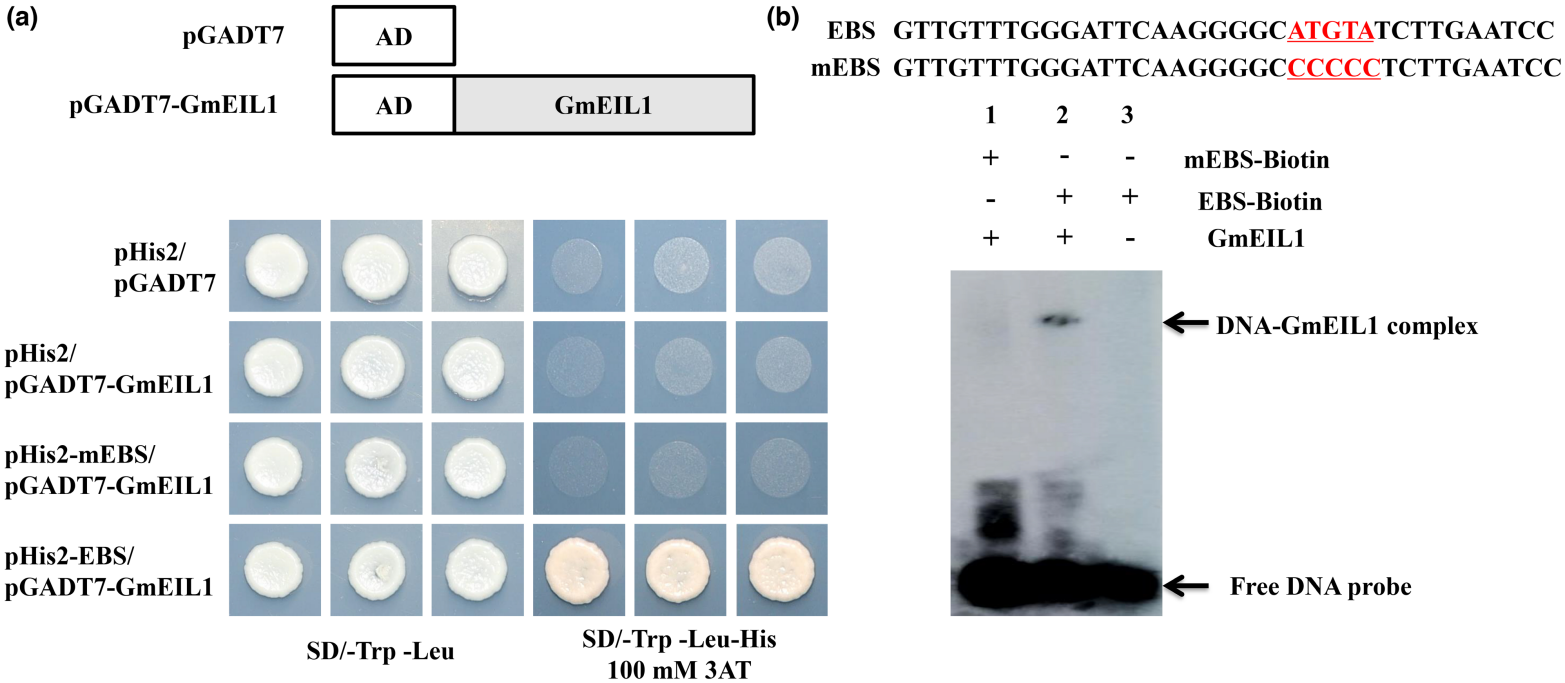
GmEIL1 binds to the EIN3‐binding sequence (EBS). (a) Yeast one‐hybrid assay for the interaction between GmEIL1 and EBS. The EBS or the mutated EBS was used as bait. Yeast cells were spotted onto selective medium (SD/−Leu/−Trp/−His) containing 100 mM 3‐amino‐1,2,4‐triazole (bottom). Cells containing pHis2 co‐transformed with pGADT7 (top row) and pHis2 co‐transformed with pGADT7‐GmEIL1 (second row) were used as negative controls. Each transformation used two different colonies for each pairwise interaction test. (b) Nucleotide sequences of the biotin‐labelled EBS and mEBS probes used for the electrophoretic mobility shift assay to analyse GmEIL1 protein binding to the EBS. 36‐bp fragments containing either the EBS or the mEBS were labelled with digoxigenin‐ddUTP and used as the probes.

To identify potential downstream target genes of GmEIL1, we first tested whether GmEIL1 could bind to an EBS using a typical EBS and a mutated EBS (mEBS, Figure 5b). In a yeast one‐hybrid assay, yeast cells co‐transformed with pHis2‐EBS + pGADT7‐GmEIL1 grew well on SD/−Trp/−Leu/−His/+3AT plates (Figure 5a), indicating that GmEIL1 bound specifically to the EBS in yeast cells. In contrast, no growth was observed in cells cotransformed with pHis2‐mEBS + pGADT7‐GmEIL1 (mutated EBS), pHis2 (empty reporter vector control) + pGADT7‐GmEIL1 or pHis2 + pGADT7 (double empty vector control), indicate no binding. We further investigated the ability of GmEIL1 to bind to the EBS in an electrophoretic mobility shift assay (EMSA) using biotin‐labelled EBS and mEBS nucleotide sequences as probes (Figure 5b). Both the yeast one‐hybrid and the EMSA results showed that GmEIL1 recognized and specifically bound to the EBS (Figure 5b, lane 2), but not to the mEBS (Figure 5b, lane 1).

**FIGURE 6:**
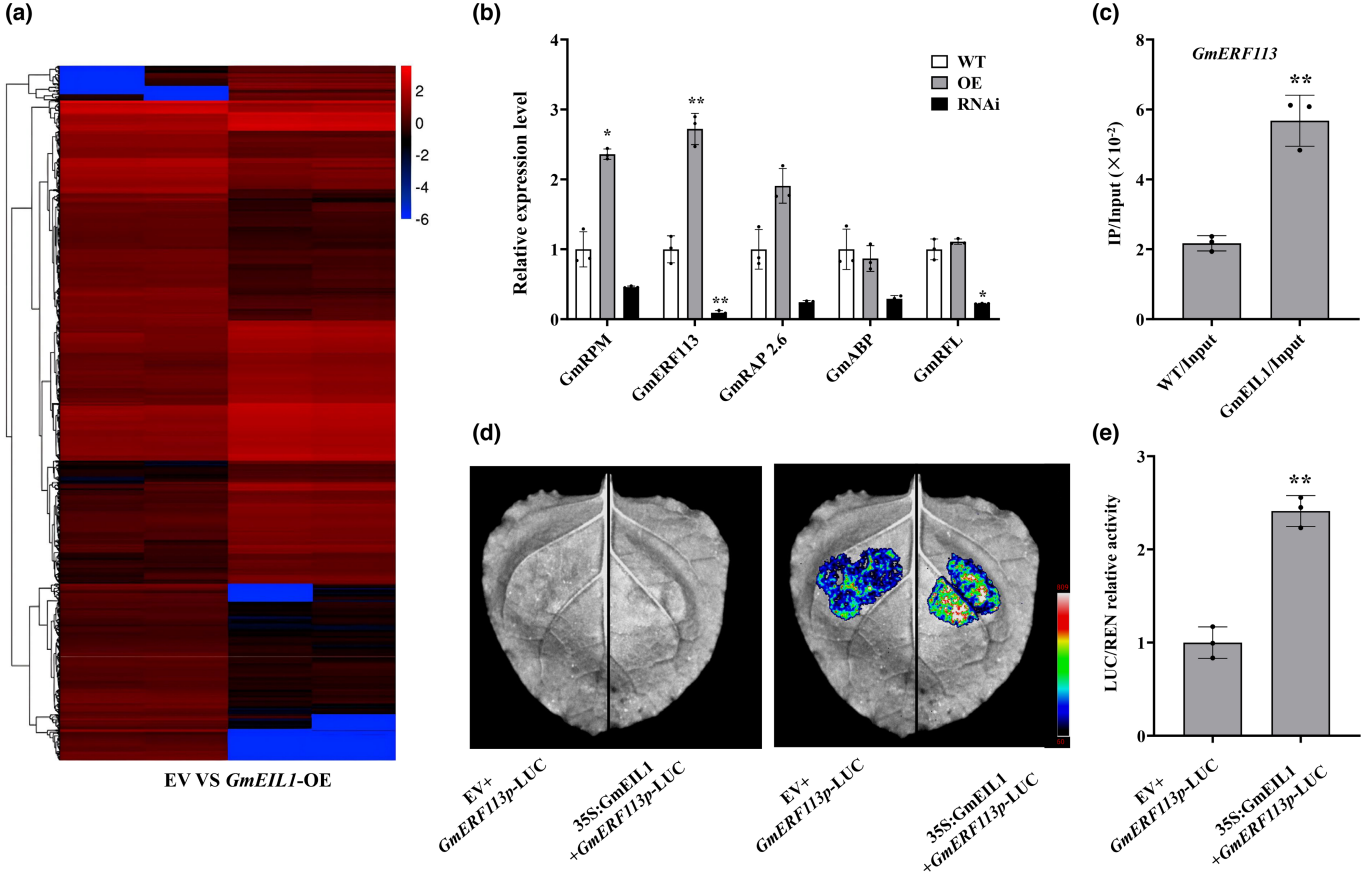
GmEIL1 positively regulates *GmERF113* expression. (a) Heatmap of genes significantly differentially expressed (*p* < 0.01) between empty vector (EV)‐containing controls and *GmEIL1*‐OE transgenic soybean hairy root lines, as determined by RNA‐sequencing analysis. The colour key indicates the fold‐changes as log_2_ values. (b) Relative expression levels of genes downstream of *GmEIL1* in *GmEIL1‐*OE or *GmEIL1*‐RNAi soybean plants. *GmEF1b* was used as an internal control to normalize all data. The experiment was performed using three biological replicates, each with three technical replicates. Data were analysed using Student's *t* test (*p < 0.05, **p < 0.01) compared with the wild type (WT, Dongnong 50). Error bars indicate the standard errors of the means. (c) Chromatin immunoprecipitation‐quantitative PCR analysis of GmEIL1 binding to the *GmERF113* promoter. Precipitated chromatin fragments were analysed by quantitative PCR using a primer targeted upstream of *GmERF113*. One‐tenth of the input chromatin (without antibody precipitation) was used as a control. Data represent the means of three biological replicates, each with three technical replicates, and were analysed using Student's *t* test (**p < 0.01). Error bars indicate standard errors of the means. (d) Luciferase (LUC) signals in the presence of empty vector (EV) or GmEIL1 in *Nicotiana benthamiana* leaves as revealed by dual‐luciferase assays. (e) Quantification of LUC/REN activity shows that GmEIL1 activated the transcription of *GmERF113*. The combination of the reporter construct (*GmERF113p*‐LUC) and the empty vector construct (EV) was used as the control. Data represent the means of three biological replicates, each with three technical replicates, and were analysed using Student's *t* test (**p < 0.01). Error bars indicate standard errors of the means.

Then, we used RNA sequencing (RNA‐seq) analysis to study the regulation of potential downstream target genes of GmEIL1 in *GmEIL1*‐OE hairy roots. With the false‐discovery rate set at <0.01 and fold‐change set at >2, we identified 1420 differentially expressed genes (DEGs) between empty vector‐transformed and *GmEIL1*‐OE hairy roots. Of these, 646 were significantly up‐regulated and 774 were significantly down‐regulated in the *GmEIL1*‐OE hairy roots (Figures 6a and S7a).

Gene ontology analysis indicated that these DEGs participate in multiple biological processes, cellular components, and molecular functions. Among them, biological processes with significant enrichment included immune system process, growth, signalling, multi‐organism process, localization, biological regulation, and response to stimulus. Cellular components with significant enrichment included extracellular matrix, extracellular region part, extracellular region, cell junction, membrane part, and membrane. Molecular functions with significant enrichment included guanyl‐nucleotide exchange factor activity, nutrient reservoir activity, antioxidant activity, receptor activity, electron carrier activity, molecular transducer activity, nucleic acid binding transcription factor activity, and catalytic activity (Figure S7b).

We screened for several genes associated with stress responses in up‐regulated DEGs based on RNA‐seq data analysis. Among the genes with EBS sequences in their promoters were *GmERF113* (Glyma.16G047600), *GmRPM* (Glyma.09G210400), *GmRAP2.6* (Glyma.20G168500), *GmABP* (Glyma.17G049800), and *GmRFL* (Glyma.08G119200). We used RT‐qPCR to measure the transcript levels of these EBS‐containing genes (*GmERF113*, *GmRPM*, *GmRAP2.6*, *GmABP*, and *GmRFL*) in *GmEIL1*‐OE and *GmEIL1*‐RNAi hairy roots. The relative expression level of *GmRAP2.6* and *GmABP* was not significantly different between transgenic soybean hairy roots and those of empty vector (EV) controls, and the expression level of *GmRPM* was higher in *GmEIL1*‐OE hairy roots compared with the EV control, but not significantly different from that in *GmEIL1*‐RNAi. The expression level of *GmRFL* was lower in *GmEIL1*‐RNAi hairy roots compared with the EV control, but not significantly different from that in *GmEIL1*‐OE. The relative expression level of *GmERF113* was significantly increased in *GmEIL1*‐OE hairy roots compared with the EV control. However, in *GmEIL1*‐RNAi hairy roots, *GmERF113* showed dramatically reduced expression (Figure 6b). Using chromatin‐immunoprecipitation (ChIP)‐qPCR analysis, we verified that GmEIL1 bound directly to the EBS of the *GmERF113* promoter (Figure 6c), and that GmEIL1 did not bind to the EBS in the promoter of *GmRPM*, *GmRAP2.6*, *GmABP*, or *GmRFL* (Figure S8).

To determine whether GmEIL1 regulates the expression of *GmERF113*, we used the luciferase expression system in *Nicotiana benthamiana* leaves. A luciferase gene under control of the *GmERF113* promoter (*GmERF113p*‐LUC) was co‐infiltrated into leaves with *35S*:*GmEIL1*. We found that GmEIL1 increased the activity of the *GmERF113* promoter (Figure 6d). Detection of LUC/REN activity showed that the activity of 35S:GmEIL1 + *GmERF113p*‐LUC was 2.27 times higher than that of the control, indicating that GmEIL1 activated *GmERF113* transcription (Figure 6e). These results demonstrated that *GmERF113* is a downstream target gene directly regulated by GmEIL1.

### 2.5 GmEIL1 is a positive regulator of ethylene‐dependent signalling during the response to *P. sojae*

To test whether GmEIL1 enhances soybean resistance to *P. sojae* through the ethylene pathway, we first tested if ethylene affects the resistance of soybean to *P. sojae*. The accumulation of *P. sojae* in the ethylene‐treated roots or cotyledons of WT (Dongnong 50) plants was significantly less than that in the non‐ethylene‐treated WT plants (Figure S9). Treatment with ethylene enhanced resistance to *P. sojae*, and accumulation of *P. sojae* in the ethylene‐treated roots or cotyledons of *GmEIL1*‐OE plants was significantly less than in the non‐ethylene‐treated *GmEIL1*‐OE plants. Compared with the roots or cotyledons of non‐ethylene‐treated *GmEIL1*‐RNAi plants, the accumulation of *P. sojae* in the roots or cotyledons of ethylene‐treated *GmEIL1*‐RNAi plants was somewhat less but not significantly different (Figure S9). Therefore, our results showed that GmEIL1 involvement in the ethylene pathway enhances soybean resistance to *P. sojae*.

**FIGURE 7:**
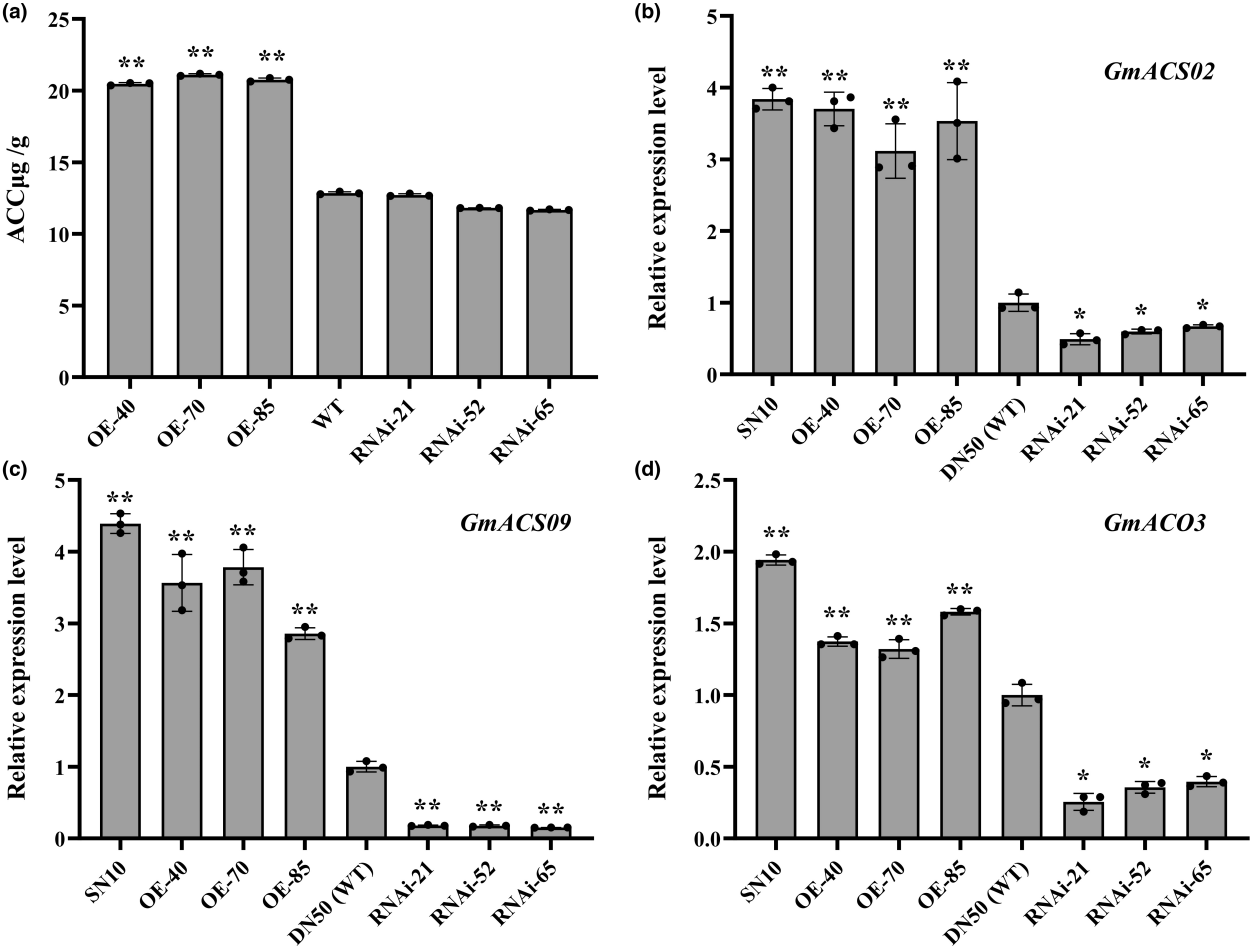
Relationship between *GmEIL1* and ethylene biosynthesis in soybean. (a) Aminocyclopropane‐1‐carboxylic acid (ACC) content in leaves of *GmEIL1‐*OE, *GmEIL1*‐RNAi, and wild type (WT, Dongnong 50) plants. (b–d) The relative transcript levels of ethylene biosynthesis‐related genes *GmACS02* (b), *GmACS09* (c) and *GmACO3* (d) in Suinong 10 (SN10), and Dongnong 50 (DN50) WT and *GmEIL1* transgenic soybean plants. *GmEF1b* (NM_001248778) was used as the internal control to normalize all data. Statistical analysis in (a–d) was performed using three biological replicates, each with three technical replicates. Data were analysed using Student's *t* test (*p < 0.05, **p < 0.01). Error bars indicate the standard errors of the means.

To further investigate how *GmEIL1* enhances soybean resistance to *P. sojae* through the ethylene pathway, we analysed genes involved in ethylene biosynthesis and ethylene accumulation. To determine whether GmEIL1 is involved in resistance to *P. sojae* by regulating ethylene accumulation, we analysed the content of ACC, the direct precursor of ethylene, in *GmEIL1* transgenic soybean plants. In addition, we measured the expression of *GmACS02* (Glyma.16G032200), *GmACS09* (Glyma.09G255000), and *GmACO3* (Glyma.02G268400), which encode key enzymes in ethylene biosynthesis. As shown in Figure 7a, the ACC content in *GmEIL1*‐OE leaves was significantly higher than the ACC content in WT leaves. However, there was no significant difference in the ACC content between WT and *GmEIL1*‐RNAi plants. The transcript levels of *GmACS02*, *GmACS09*, and *GmACO3* were markedly higher in *GmEIL1*‐OE plants than in WT and *GmEIL1*‐RNAi plants (Figure 7b–d). Together, these results indicated that GmEIL1 plays a role in regulating ethylene accumulation.

To investigate whether GmEIL1 activates gene expression by binding to the promoters of genes involved in ethylene biosynthesis (*GmACS02*, *GmACS09*, and *GmACO3*), we performed a luciferase reporter assay. GmEIL1 bound to the *GmACS09* promoter and thus directly activated its expression, while GmEIL1 did not bind to the promoters of *GmACS02* and *GmACO3* (Figure S10). Subsequently, we monitored the expression pattern of *GmEIL1* in *GmEIL1* transgenic soybean plants following ethylene treatment. *GmEIL1* expression was induced by ethylene in the leaves of *GmEIL1*‐OE plants, reaching its highest value at 9 h, then dropping sharply. In contrast, in the *GmEIL1*‐RNAi leaves, there was not a significant change in *GmEIL1* transcript abundance following the ethylene treatment, demonstrating a difference in *GmEIL1* expression between *GmEIL1*‐OE and *GmEIL1*‐RNAi plants (Figure S11).

We also measured the ACC content in the roots and cotyledons of *P. sojae‐*infected transgenic plants. The ACC content was significantly higher in *P. sojae*‐infected *GmEIL1*‐OE plants than in uninfected *GmEIL1*‐OE plants (Mock), and it was significantly higher in *P. sojae*‐infected WT (Dongnong 50) plants than in uninfected WT plants (Mock). This suggested that the ACC content contributes to soybean resistance against *P. sojae*, and that GmEIL1 increases the ACC content, leading to enhanced resistance to *P. sojae* (Figure S12). These results indicated that GmEIL1 enhances soybean resistance to *P. sojae* by regulating ethylene accumulation. We also analysed the expression patterns of *GmACS02*, *GmACS09*, and *GmACO3* in response to *P. sojae* in the resistant cultivar Suinong 10 and the susceptible cultivar Dongnong 50 (Figure S13). The result indicated that the expression levels of *GmACS02*, *GmACS09*, and *GmACO3* in Suinong 10 or Dongnong 50 correlated with the resistance levels of these two lines. Taken together, these results suggested that *GmEIL1* promotes ethylene accumulation and ethylene biosynthesis genes expression in response to *P. sojae* infection.

### 2.6 GmEIL1 regulates the expression of genes in the ethylene signal transduction pathway in response to *P. sojae* infection

**FIGURE 8:**
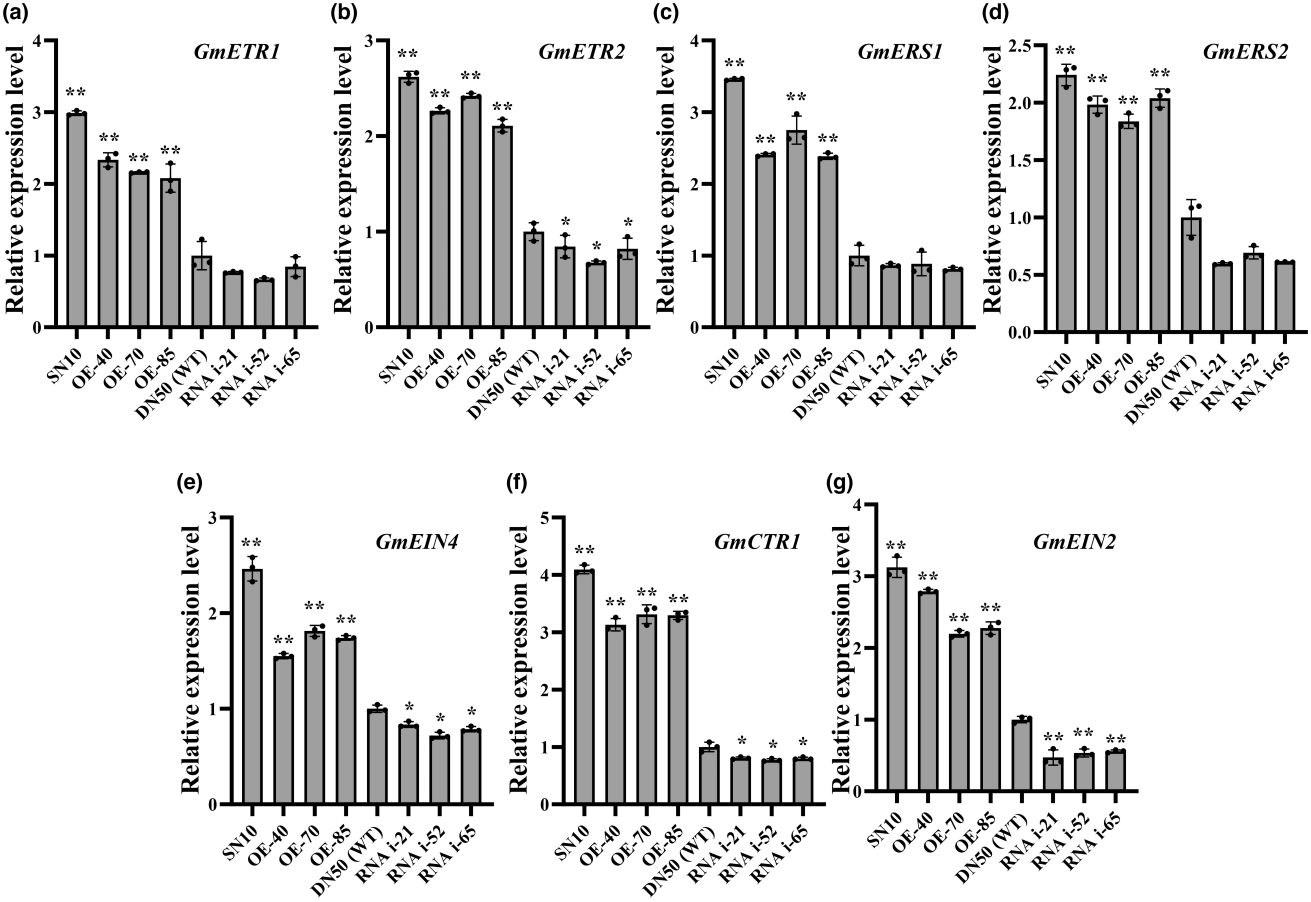
Relative expression levels of ethylene pathway‐related genes in *GmEIL1* transgenic plants. Relative transcript abundance of *GmETR1*, *GmETR2*, *GmERS1*, *GmERS2*, *GmCTR1*, *GmEIN4*, and *GmEIN2* in *GmEIL1*‐OE and *GmEIL1*‐RNAi plants, and in Suinong 10 (SN10) and Dongnong 50 (DN50) wild‐type (WT) plants. *GmEF1b* was used as the internal control to normalize all data. Statistical analyses in (a–g) were performed on three biological replicates, each with three technical replicates. Data were analysed using Student's *t* test (*p < 0.05, **p < 0.01). Error bars indicate the standard errors of the means.

*AtEIL1* is associated with ethylene biosynthesis and with the ethylene signal transduction pathway (An et al., 2010). To further analyse whether *GmEIL1* is involved in the ethylene signal transduction pathway that modulates soybean resistance to *P. sojae*, we measured the expression of several genes in the ethylene signal transduction pathway: *GmETR1* (EF210138), *GmERS1* (EF210137), *GmETR2* (EF210139), *GmERS2* (XM_028366968), *GmEIN4* (EF210140), *GmCTR1* (XM_003542487), and *GmEIN2* (XM_006588735). The transcript levels of these genes were significantly higher in Suinong 10 and *GmEIL1*‐OE plants than in Dongnong 50 and *GmEIL1*‐RNAi plants (Figure 8a–g). The results suggest that *GmEIL1* is involved in soybean resistance to *P. sojae* through the ethylene signal transduction pathway.

To investigate whether GmEIL1 activates gene expression by directly binding to the promoters of the ethylene signal transduction pathway‐related genes analysed above, we performed a luciferase reporter assay and found that GmEIL1 did not bind to the promoters of these genes, and thus did not appear to directly activate their expression (Figure S14). We also analysed the expression patterns of several ethylene signal transduction pathway genes in the response to *P. sojae* in the resistant cultivar Suinong 10 and the susceptible cultivar Dongnong 50. The expression levels of ethylene signal transduction pathway genes in Suinong 10 or Dongnong 50 were correlated with the resistance levels of these two cultivars (Figure S15). Taken together, these results suggested that GmEIL1 promotes ethylene signal transduction pathway‐related genes expression in response to *P. sojae* infection.

### 2.7 Expression of *GmPR1* was increased in the *GmEIL1*‐OE plants

Pathogenesis‐related (PR) proteins are important for plant defence responses to pathogens (Van Loon et al., 2006; Van Loon & Van Strien, 1999). In a previous study, we found that GmERF113 positively regulates the expression of *PR1*, which enhances soybean resistance to *P. sojae* (Zhao et al., 2017). To investigate whether *GmPR1* expression is involved in GmEIL1‐regulated resistance to *P. sojae*, we measured the transcript levels of *GmPR1* (XM_003545722) in the *GmEIL1*‐OE and *GmEIL1*‐RNAi plants. *GmPR1* expression was increased in *GmEIL1*‐OE plants compared with that in WT plants. However, in *GmEIL1*‐RNAi plants, *GmPR1* expression was dramatically reduced (Figure S16), indicating that GmEIL1 improves soybean defence against *P. sojae* by enhancing *GmPR1* expression. Ethylene also induced the expression of *GmPR1* (Figure S17). GmEIL1 promotes *GmPR1* expression in response to *P. sojae* infection.

## 3 DISCUSSION

A variety of genes have been reported to be involved in the response of soybean to *P. sojae* infection (Cheng et al., 2018; Gao et al., 2022; Jahan et al., 2020; Wang, Wang, et al., 2019; Zhang et al., 2021). Functional studies of these genes will help us elucidate the genetic mechanisms of soybean defence against *P. sojae* (Jing et al., 2016; Kong et al., 2015; Xu et al., 2014; Zhang, Gao, et al., 2019). In a previous study, we used a suppressive subtractive hybridization library and cDNA microarrays to identify a highly up‐regulated *EIN3* homologue in resistant Suinong 10 soybean plants that were infected with *P. sojae* (Xu et al., 2012). In this study, to further investigate the mechanism of soybean resistance to *P. sojae*, we isolated *GmEIL1*, a member of the *EIN3/EIL* gene family, from the resistant cultivar Suinong 10 and showed that it improved soybean resistance to *P. sojae*. *EIN3* was first isolated from the *Arabidopsis* ethylene‐insensitive mutant (*ein3*) by Chao et al. (1997) using transposon tagging. Similar to other EIN3 proteins, GmEIL1 has a 125‐amino acid EIN3 domain, signifying that it is a member of the EIN3/EILs family.

Functional roles of EIN3/EILs family transcription factors and the underlying mechanisms have been studied in several plants (Chao et al., 1997; Huang et al., 2010; Waki et al., 2001). EIN3/EILs family members are involved in pathogen responses in a variety of plants (Alonso et al., 2003; Xu et al., 2009). In *Arabidopsis*, concurrent loss of functional WEI5, EIL1, and EIN3 nearly completely abolishes the ethylene response in etiolated seedlings, and adult plants are highly susceptible to infection by the necrotrophic fungal pathogen *Botrytis cinerea* (Alonso et al., 2003). Following inoculation of a highly resistant oilseed rape (*Brassica napus*) variety with *Sclerotinia sclerotiorum*, *BnEIN3* expression levels were significantly higher than levels in moderately resistant or susceptible varieties, indicating that *BnEIN3* plays an important role in resistance to *S. sclerotiorum* (Xu et al., 2009). When *GhEIN3* was silenced in cotton (*Gossypium hirsutum*) plants subjected to a *Fusarium oxysporum* f. sp. *vasinfectum* inoculation test, the *GhEIN3*‐silenced plants were more susceptible to the pathogen than control plants, demonstrating that *GhEIN3* plays a positive role in cotton resistance to Fusarium wilt (Zhao et al., 2022). Likewise, we showed here that *GmEIL1* expression significantly increased following *P. sojae* infection. To further examine the molecular basis of the *GmEIL1* response to *P. sojae*, we examined the *P. sojae* resistance in transgenic soybean plants engineered to have enhanced (OE) or suppressed (RNAi) expression of *GmEIL1* and showed that *GmEIL1* enhanced soybean resistance to *P. sojae*.

EIN3 and EIL proteins can directly bind to the promoters of target genes to activate their transcription in plant growth and development processes as well as in response to biotic and abiotic stresses (Chen et al., 2023; Dolgikh et al., 2019; Peng et al., 2014; Wang, Xu, et al., 2019; Zhang et al., 2011). In *Hevea brasiliensis*, potential HbEIN3 target genes were identified, including latex biosynthesis and drainage‐related genes (Wang, Xu, et al., 2019). Some transcription factor genes that are targets of EIN3, including ERF, PIF3, RSL4, ESE1, and CBF1/2/3, also play important roles in yellowing, root hair development, and salt and cold stress responses in *Arabidopsis* (Dolgikh et al., 2019). In tomato, SlEIN3 functions in modulating β‐carotene and ascorbic acid levels by directly regulating *SlERF.H30* and *SlERF.G6* (Chen et al., 2023). Also, *Arabidopsis* EIN3 specifically binds the promoters of the salt‐related genes *ESE1* and *SIED1* to regulate their expression and increase salt tolerance (Peng et al., 2014; Zhang et al., 2011).

In this study, we screened for target genes of GmEIL1 and confirmed that *GmERF113* is a target gene directly regulated by GmEIL1. GmEIL1 activated *GmERF113* transcription by directly binding to its promoter. Some members of the EIN3/EILs family bind directly to the promoters of genes and repress their transcription (Shi et al., 2012; Song et al., 2022; Yokotani et al., 2009). For example, EIN3 negatively regulates ethylene biosynthesis and ethylene signal‐mediated freezing tolerance in *Arabidopsis* by directly binding to the promoters of cold‐inducible *CBF* and type‐A *ARR* genes and suppressing their expression (Shi et al., 2012). In *Betula platyphylla*, BpEIN3.1 inhibits the transcription of *BpATPS1* by binding to its promoter (Song et al., 2022). In tomato, transcription of *SlACS2* and *SlACS4* is suppressed by SlEIL (Yokotani et al., 2009). This is in contrast to our findings that GmEIL1 activates target gene transcription. Therefore, it is evident that EIN3/EILs family members play different roles in different plants and are involved in a variety of biological processes and stresses.

We previously reported that *GmERF113* expression is induced by ethylene, and overexpressing *GmERF113* improves soybean resistance to *P. sojae* (Zhao et al., 2017). Our current results revealed increased *GmERF113* transcript levels in transgenic soybean plants overexpressing *GmEIL1*, indicating that GmEIL1 positively regulates the expression of *GmERF113*. Therefore, a GmEIL1–GmERF113 module may be involved in soybean resistance to *P. sojae*. Moreover, Zhao et al. (2017) reported that GmERF113 regulates the expression of *PR1* in soybean, which decreased the infection of soybean by *P. sojae*. We therefore analysed the expression of *GmPR1* in *GmEIL1* transgenic soybean plants in this study. Our results showed that *GmPR1* expression increased in *GmEIL1*‐OE plants compared to WT plants, suggesting that *GmPR1* was activated, thus *GmEIL1* enhances soybean resistance to *P. sojae*, possibly due to increased levels of GmPR1. In addition, the GmEIL1–GmERF113 cascade regulating *GmPR1* expression may also be involved in soybean resistance to *P. sojae*.

Ethylene plays a key role in regulating plant responses to pathogen attack and environmental stresses (Broekaert et al., 2006; Geraats et al., 2003; Hoffman et al., 1999; Liang et al., 1996; Seo et al., 2010). For example, Singh et al. (2004) showed that ethylene biosynthesis in rice induced by flood or hypoxia is essential for mediating resistance to rice blast infection. This research also showed that the severity of blast disease increased following treatment with amino‐ethoxyvinylglycine hydrochloride, an ethylene biosynthesis inhibitor, and eliminated the flood‐induced resistance in the rice plants. In contrast, rice blast resistance in susceptible varieties was significantly enhanced following treatment with ethephon (2‐chloroethylphosphonic acid), an ethylene‐generating compound. *OsACS1*, *OsACS2* and *OsACO7* were significantly induced upon infection with *M. oryzae* (Iwai et al., 2006). In another study, silencing of *OsACS2* and *OsACO7* resulted in increased rice susceptibility to rice blast (Seo et al., 2010). The *OsACS2*‐OE lines showed significantly increased levels of *OsACS2* transcripts, endogenous ethylene, and defence gene expression, especially in response to infection with *M. oryzae* or the sheath blight fungus *Rhizoctonia solani*. This suggests that pathogen‐inducible production of ethylene in transgenic rice can enhance resistance to necrotrophic and hemibiotrophic fungal pathogens (Helliwell et al., 2013).

In this study, the mRNA transcripts of *GmEIL1* increased remarkably due to ethylene treatment. Furthermore, the ethylene content and the transcript levels of ethylene biosynthesis‐related genes *GmACS02*, *GmACS09*, and *GmACO3* in *GmEIL1*‐OE plants were significantly higher than those of WT and *GmEIL1*‐RNAi plants, suggesting that *GmEIL1* is involved in ethylene biosynthesis. This strongly suggests that GmEIL1 participates in *P. sojae* resistance by enhancing ethylene biosynthesis. In addition, compared with WT plants, the ACC content decreased slightly but not significantly in *GmEIL1*‐RNAi plants. At the same time, the biomass accumulation of *P. sojae* in *GmEIL1*‐RNAi plants decreased following ethylene treatment, but it was not significant compared with untreated *GmEIL1*‐RNAi plants. We surmise that this may be due to the following two points. First, *GmEIL1*‐RNAi may not have silenced *GmEIL1* homologues (*GmEIL1c* and *GmEIL1d*), which may also promote ACC and ethylene biosynthesis and regulate *P. sojae* resistance. Second, RNAi‐mediated gene silencing can have off‐target effects (Fedorov et al., 2006; Kobayashi et al., 2022). Therefore, we speculate that the silencing efficiency of *GmEIL1*‐RNAi may not be 100% due to potential off‐target effects. This could affect the nonspecific expression of some genes, and these genes may also affect ACC biosynthesis, which may enhance *P. sojae* resistance through the ethylene pathway.

We also studied the expression of genes involved in the ethylene signal transduction pathway, i.e., *GmETR1*, *GmERS1*, *GmETR2*, *GmERS*, *GmEIN4*, *GmCTR1*, and *GmEIN2*, and found that the relative expression of these genes was markedly higher in *GmEIL1*‐OE plants than in WT plants. ERFs play important roles in regulating tolerance to biotic and abiotic stresses in plants. For example, in Chinese cabbage (*Brassica rapa* subsp. *pekinensis*), overexpression of the *BrERF11* transcription factor gene enhances disease resistance to the bacterial wilt pathogen *Ralstonia solanacearum* (Lai et al., 2013). *AtERF5* positively regulates plant defence against the bacterial pathogen *P. syringae* pv. *tomato* DC3000, which causes bacterial brown spot (Son et al., 2012). Our previous study suggested that *GmERF113* expression depends primarily on ethylene signalling pathways, which mediate soybean responses to *P. sojae* infection (Zhao et al., 2017). Sugano et al. (2013) also reported ethylene‐induced resistance to *P. sojae* in soybean. In this study, *GmEIL1* was significantly induced by ethylene, and GmEIL1 directly regulated the expression of *GmERF113*, strongly suggesting involvement in soybean disease resistance via the ethylene signalling transduction pathway. However, how GmEIL1 can simultaneously exert its regulatory function in ethylene biosynthesis and signal transduction pathways remains to be further studied.

**FIGURE 9:**
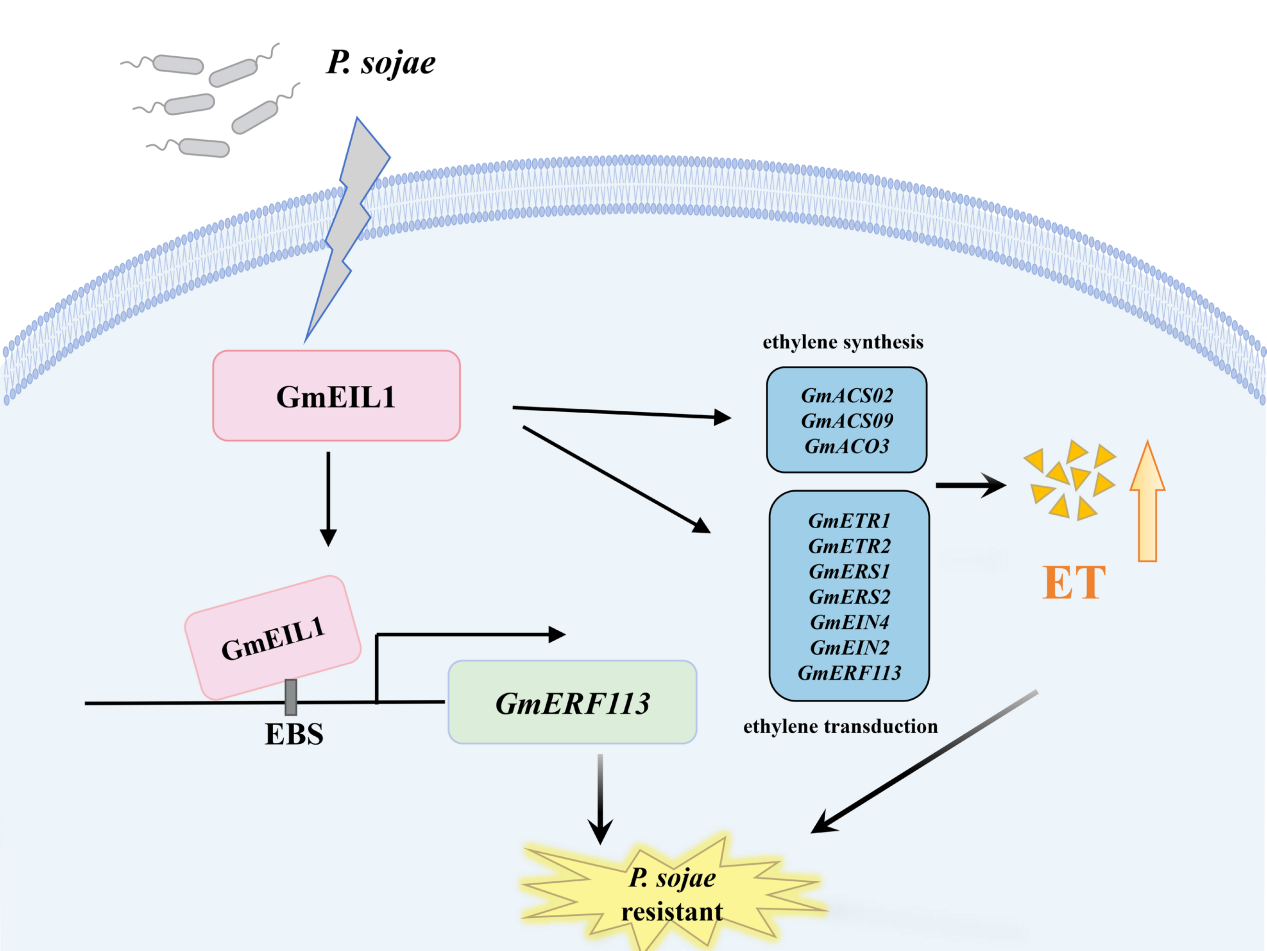
A proposed model for the molecular mechanism by which GmEIL1 and GmERF113 regulate soybean resistance to *Phytophthora sojae*. *GmEIL1* is stimulated by *P. sojae* infection, and GmEIL1 enhances *GmERF113* expression by binding to the EIN3‐binding sequence (EBS) element in the *GmERF113* promoter, thus activating the soybean defence response to *P. sojae*. In addition, GmEIL1 promotes the accumulation of ethylene by activating the expression of genes related to ethylene biosynthesis and ethylene transduction pathway genes to enhance resistance to *P. sojae*. ET, ethylene; the yellow triangle represents ethylene produced.

Based on these results, we constructed a model proposing how GmEIL1 participates in *P. sojae* resistance via an ethylene‐mediated disease resistance network (Figure 9). According to our model, *GmEIL1* is stimulated by *P. sojae* infection, and *GmEIL1* enhances *GmERF113* expression by binding to the EBS element in the *GmERF113* promoter, thus activating the soybean defence response to *P. sojae*. In addition, GmEIL1 promotes the accumulation of ethylene by activating the expression of genes related to ethylene biosynthesis and ethylene transduction pathway genes to enhance resistance to *P. sojae*.

## 4 EXPERIMENTAL PROCEDURES

### 4.1 Plant materials and stress treatments

Seeds of the soybean (*Glycine max*) cultivars Suinong 10 (highly resistant to race 1 of *P. sojae*) (Zhang et al., 2010) and Dongnong 50 (highly susceptible to race 1 of *P. sojae*; obtained from the Key Laboratory of Soybean Biology at the Chinese Ministry of Education, Harbin) were germinated and grown in vermiculite at 22°C and 70% relative humidity with a 16 h light/8 h dark photoperiod in a glasshouse. The soybean cultivar Dongnong 50 was used for genetic transformation experiments. *A. thaliana* plants used for subcellular localization were grown in a growth chamber at 22°C with an 8 h light/16 h dark photoperiod. *Nicotiana benthamiana* plants for luciferase assays were grown in a growth chamber at 22°C under a 16 h light/8 h dark photoperiod (Fehr et al., 1971). Suinong 10 and Dongnong 50 seedlings at the first node stage (V1) (Fehr et al., 1971) were used for ethylene treatments, which were performed in sealed plexiglass chambers by applying a solution of 2 mL of 40% Ethephon and 1 g of NaHCO_3_ dissolved in 200 mL water. Leaves were sampled at 0, 1, 3, 6, 9, 12, and 24 h after initiation of ethylene treatments. *P. sojae* race 1 was obtained from the Key Laboratory of Soybean Biology in the Chinese Ministry of Education, Harbin, China. For the *P. sojae* infections, the hypocotyls of soybean cultivars Suinong 10 and Dongnong 50 were inoculated at the V1 stage using zoospores of *P. sojae* or mock inoculated with sterile water following the procedure described by Kaufmann and Gerdemann (1958), with minor modifications. The *P. sojae* zoospores were induced as described by Ward et al. (1979), and the concentration of zoospores was estimated to be about 10^5^ spores/mL using a haemocytometer. The leaves of the inoculated plants were harvested and immediately frozen in liquid nitrogen at 0, 3, 6, 9, 12, 24, 36, 48, and 72 h after the treatment and stored at −80°C until required for RNA isolation.

### 4.2 Isolation and sequence analysis of *GmEIL1*

An NCBI BLAST search (http://blast.ncbi.nlm.nih.gov/Blast.cgi) was used to identify a soybean expressed sequence tag (EST) sequence highly homologous to *EIN3*. The full‐length cDNA was designated *GmEIL1* (GenBank accession no. XM_003545435) and was amplified from Suinong 10 by RT‐PCR using the primers GmEIL1F and GmEIL1R (Table S1). Cycling conditions were as follows: 94°C for 5 min; 30 cycles of 94°C for 30 s, 55°C for 30 s, and 72°C for 90 s; followed by 72°C for 10 min; then held at 4°C. The amplification product was constructed into the pMD18‐T vector (TaKaRa) and sequenced for confirmation. Analysis of protein structure was performed using Interpro scan (http://www.ebi.ac.uk/interpro/scan.html), and the three‐dimensional structure was predicted using InterPro AlphaFold (https://www.ebi.ac.uk/interpro/). Sequence alignments were performed using DNAMAN software. A phylogenetic analysis of *GmEIL1* and homologous *EIN3* family members was performed using MEGA 5.1 software.

### 4.3 RT‐qPCR

Total RNA was isolated from soybean leaves by adding 1 mL TRIzol reagent (Invitrogen) according to the manufacturer's instructions, and then used as the template for first‐strand cDNA synthesis. qPCR analysis was performed on samples from various treatments on a LightCycler 96 Touch Real‐time PCR Detection system (Roche). One microlitre of diluted (1:10, vol/vol) first‐strand cDNA was used as template in a 20‐μL reaction volume. The transcript levels were normalized to *GmEF1b* (GenBank accession no. NM_001248778) levels using the 2^−ΔΔ*C*t^ method. First, the mean value of *C*_t_ of the internal reference gene in each group was calculated, and the first Δ*C*_t_ was calculated, then the value for the target gene to be examined in each group was subtracted from the *C*_t_ value of the internal reference gene. Subsequently, the mean value of Δ*C*_t_ for the control group was calculated, and then each Δ*C*_t_ was subtracted from the mean value of Δ*C*_t_ of the control group just calculated to obtain ΔΔ*C*_t_ and finally the relative expression was calculated as (2^−ΔΔ*C*t^). The statistical analyses were performed using the software GraphPad Prism v. 8.00. Student's *t* test was used to test the significance of difference for single‐pair samples. Each analysis was performed on three biological replicates, each with three technical replicates.

### 4.4 Subcellular localization

The full‐length coding region of *GmEIL1* was fused to sequences encoding the N‐terminus of GFP under the control of the cauliflower mosaic virus 35S promoter (35S) in the pCAMBIA1302 vector using primers GmEIL1‐GF and GmEIL1‐GR (Table S1). The GmEIL1‐GFP and H2B‐mCherry fusion plasmids (or 35S:GFP and the H2B marker (H2B, NM_122194.3) for the nucleus) were cotransformed into *Arabidopsis* protoplasts using a polyethylene glycol‐mediated method (Yoo et al., 2007). Localization of the fusion proteins was visualized using a TCS SP2 confocal spectral microscope imaging system (Leica). The transfected protoplasts were incubated in weak light at 22°C for 16–20 h. Protoplasts were collected and lysed to extract total proteins. For localization of GmEIL1‐GFP or GFP, nuclei were isolated using a Protein Isolate Extraction Kit (Sangon Biotech). The supernatants of extracts were separated by SDS‐PAGE. After electrophoresis, the proteins were transferred to polyvinylidene difluoride membranes (Millipore) and probed using anti‐GFP antibodies (Abmart). Signals on immunoblots were quantified using ImageJ software.

### 4.5 EMSA

DNA‐binding activity of GmEIL1 was evaluated by EMSA according to the method of Garner and Revzin (1981). Digoxigenin‐ddUTP‐labelled double‐stranded oligonucleotides representing EBS and mEBS (Figure 5b) were used as the probes. Recombinant GmEIL1 was purified using a His‐Bind Kit (EMD Millipore) according to the manufacturer's protocol. Signals were detected by chemiluminescence and recorded on an ImageQuant LAS 500 (GE).

### 4.6 Yeast one‐hybrid assay

To further analyse the ability of GmEIL1 to bind to the EBS, GmEIL1 was cloned into the GAL4 activation vector pGADT7 and the EBS (ATGTA) or mutated EBS (mEBS, CCCCC, Figure 5b) was cloned into the pHis2 vector. Competent yeast cells (strain Y187) were prepared according to the protocol in the Epigenetics Frozen‐EZ Yeast Transformation II Kit (Zymo Research). For yeast transformation, co‐transformed cells were plated onto SD (−Trp, −Leu) or SD (−Trp, −Leu, −His) plates including 100 mM 3‐amino‐1,2,4‐triazole (3‐AT) (Sigma‐Aldrich) and incubated at 30°C for 3 days, after which the growth of yeast cells was observed.

### 4.7 Soybean transformation

The full‐length *GmEIL1* cDNA was cloned into the pCAMBIA3301 vector (www.cambia.org) using the primers GmEIL1‐oF and GmEIL1‐oR (Table S1). Amplification of two identical 466‐bp fragments of *GmEIL1* was performed using two primer pairs GmEIL1‐rF1 and GmEIL1‐rR1, and GmEIL1‐rF2 and GmEIL1‐rR2, respectively. The two fragments were attached to the same pFGC5941 vector. Recombinant plasmid pCAMBIA3301‐*GmEIL1* and recombinant plasmid pFGC5941‐*GmEIL1* were introduced into soybean Dongnong 50 via *Agrobacterium tumefaciens*‐mediated transformation as described by Paz et al. (2004). *A. tumefaciens* LBA4404 containing recombinant pFGC5941‐*GmEIL1* at a density of OD_600_ = 0.5 was used to infect the cotyledonary nodes of Dongnong 50 soybean explants. Then, the explants were cultured on shoot induction medium. Transgenic soybean plants were identified by PCR amplification and RT‐qPCR analysis, after which immunoblotting was used to identify the plants overexpressing *GmEIL1*.

### 4.8 *Agrobacterium rhizogenes*‐mediated transformation of soybean hairy roots

The full‐length *GmEIL1* cDNA sequence was cloned into the plant expression vector pCAMBIA3301. The overexpression construct was introduced into *Agrobacterium rhizogenes* K599 to generate transgenic soybean hairy roots, according to the method described by Graham et al. (2007) and Kereszt et al. (2007). The empty vector pCAMBIA3301 was used as a negative control.

### 4.9 RNA‐seq analysis

Three grams of transgenic hairy roots of *GmEIL1*‐OE and EV (empty vector) control plants were used for RNA‐seq analysis. The sequencing libraries were generated using a Next Ultra RNA Library Prep Kit for Illumina (NEB) following the manufacturer's recommendations, and index codes were added to attribute sequences to each sample. After cluster analysis, the RNA was sequenced on an Illumina Hiseq 2500 platform to generate paired‐end reads. Total reads were mapped to the soybean genome (http://www.phytozome.ne) using the Tophat tools software (Trapnell et al., 2009). Read counts for each gene were generated using HTSeq with a union mode. DEGs between samples were defined by DESeq using two separate models (Anders & Huber, 2010), based on fold change >2 and false discovery rate‐adjusted *p* < 0.01. Gene ontology enrichment analysis of the DEGs was implemented using the GOseq R packages based on Wallenius non‐central hypergeometric distribution (Young et al., 2010), which can adjust for gene length bias in DEGs.

### 4.10 Expression analysis of putative GmEIL1 target genes

*GmERF113* (GenBank accession no. XM_003548806), which has an EBS in its promoter, was identified as a putative downstream target of GmEIL1. Relative expression levels of *GmERF113* in *GmEIL1* transgenic and WT soybean plants were compared using RT‐qPCR. Expression levels of *GmEF1b* were used as an internal control.

### 4.11 ChIP‐qPCR assays

ChIP‐qPCR assays were performed as described by Saleh et al. (2008). Briefly, the sample was fixed with formaldehyde solution and chromatin was isolated and treated with ultrasound to produce DNA fragments with an average size of 500 bp. To eliminate nonspecific binding, soluble chromatin fragments were isolated and pre‐absorbed using 30 μL Protein G Plus/Protein A agarose suspension (Merck Millipore Biotechnology), then immunoprecipitated with 30 μL Protein G Plus/Protein A agarose suspension with anti‐Myc (Santa Cruz Biotechnology). Immunoprecipitation products were analysed by RT‐qPCR with SYBR Premix ExTaq Mix (Takara Bio). Data were normalized to input transcript levels and represent means from three biological replicates.

### 4.12 Detection of luciferase activity in *N. benthamiana* leaves

The methods used were based on those of Shang et al. (2010) and Song et al. (2013). Briefly, the 2.3‐kb promoter sequence of *GmERF113* (*GmERF113p*) was cloned into the pGreen II 0800‐LUC vector using primers GmERF113‐pF/R (Table S1). The reporter construct *GmERF113p*‐LUC and the effector construct *35S*:*GmEIL1* were transformed separately into *A. tumefaciens* GV3101. Transformation of *N. benthamiana* leaves by *A. tumefaciens*‐mediated infiltration was performed as described by Liu et al. (2012). Luciferase activity was observed using an automatic chemiluminescence system (Tanon 5200) at 72 h after infiltration. All primers for genotyping and vector construction are listed in Table S1.

### 4.13 Pathogen response assays of transgenic soybean plants

Live cotyledons of the T_3_ transgenic and WT soybean plants were inoculated with *P. sojae* zoospores (approximately 10^5^ spores/mL) using the methods described by Morrison and Thorne (1978) and Dou et al. (2003). Briefly, a hole with a diameter of 5 mm and a depth of 2 mm was drilled in the centre of the cotyledons. Then, 20 μL of a *P. sojae* zoospore suspension was placed into the hole as the inoculum. The concentration of the zoospore suspension was estimated to be approximately 10^5^ spores/mL. The plants were moistened with plastic film and inoculated every 6 h and cultured in a greenhouse maintained at 25°C and 100% relative humidity under a photoperiod of 16 h light/8 h dark. Inoculation of roots was performed using the procedure described by Zhang et al. (2017). Disease symptoms in cotyledons and roots were observed and photographed using a Nikon D7000 camera. Sizes of lesions on the inoculated cotyledons and roots were measured using ImageJ software (https://imagej.nih.gov/ij/index.html) according to the instructions provided. In summary, click on File‐Open in ImageJ software to open the picture that needs to be used to calculate the lesion area. First, set the appropriate scale according to the ruler on the picture. Then, click on the straight line tool in ImageJ and draw a 1 cm straight line on the ruler. Next, click on Analyse, select Set scale, set the known distance to 1, and set the unit of length to cm. After that, measure the lesion area by clicking on the polygon tool in ImageJ, circling the lesion on the plant, clicking on Analyse, and selecting Measure. The corresponding lesion area value will then appear. *P. sojae* biomass was estimated based on the transcript level of *P. sojae TEF1* (EU079791) relative to that of *GmEF1b* (NM_001248778), according to the method of Chacón et al. (2010) (primers sequences are listed in Table S1). The pathogen response assays were performed using three biological replicates, each with three technical replicates.

### 4.14 Determination of ACC contents

For ACC quantification, 0.5 g of fresh leaves of mature WT and *GmEIL1* transgenic soybean plants or 0.5 g of cotyledons and roots of *GmEIL1* transgenic and WT soybean plants inoculated with *P. sojae* were collected and ground to a powder in liquid nitrogen. Then, the ACC in each sample was extracted using acetonitrile. ACC contents were measured with high‐performance liquid chromatography (HPLC) by Suzhou Mengxi Biomedical Technology Co., Ltd., in China (Yang et al., 2014). Values presented are mean ± *SD* of three biological replicates of three individual plants.

## Supporting information


**FIGURE S1.** Sequence comparison between GmEIL1 and related EIN3/EILs family proteins. (a) Conserved EIN3 domain sequence at amino acids 174–298 of GmEIL1, EIN3, and EIL proteins. (b) Phylogenetic tree reconstructed using soybean GmEIL1, EIN3, and EIL amino acid sequences from various plant species. Amino acid sequences of 37 dirigent domains were analysed using MEGA 5.1. The source species for the EIN3 and EIL1 proteins are as follows: At, *Arabidopsis thaliana*; Gm, *Glycine max*; Pp, *Podophyllum peltatum*; Ps, *Pisum sativum*; Cm, *Cucumis melo*; Vr, *Vigna radiata*; Nt, *Nicotiana tabacum*; Vv, *Vitis vinifera*; Le, *Solanum lycopersicum*; Dc, *Dianthus caryophyllus*; Rh, *Rosa hybrida*; Lr, *Lilium regale*; Ma, *Musa acuminata*; Pl, *Paeonia lactiflora*. (c) Three‐dimensional structure of GmEIL1 and AtEIL1, with the EIN3 domain labelled.

**FIGURE S2.** Subcellular localization of GmEIL1. GmEIL1‐GFP and H2B‐mCherry fusion plasmids (or 35S:GFP and the H2B marker gene for the nucleus) were cotransformed into *Arabidopsis* protoplasts using a polyethylene glycol‐mediated method. Bright‐field images, GFP fluorescence (green), chlorophyll autofluorescence (purple), mCherry fluorescence (magenta), and the merged images are shown. Merged‐1 is a merged image of the nucleus marker control (H2B‐mCherry) and GFP channels, Merged‐2 is a merged image of all channels. Size bars indicate 10 μm. The fluorescence images indicated that GmEIL1 localizes in the nucleus.

**FIGURE S3.** Verification of *GmEIL1* overexpression and silencing in transgenic soybean plants. (a) Immunoblot analysis of *GmEIL1* expression in three overexpressing transgenic soybean lines (OE‐40, OE‐70, and OE‐85). (b) Reverse transcription‐quantitative PCR analysis of the relative expression of *GmEIL1* in *GmEIL1*‐OE, *GmEIL1*‐RNAi, and wild‐type (WT: Dongnong 50) soybean plants.

**FIGURE S4.** Expression patterns of *GmEIL1b*, *GmEIL1c*, and *GmEIL1d* in resistant cultivar Suinong 10 versus susceptible cultivars Dongnong 50. Relative expression of *GmEIL1b* (a), *GmEIL1c* (b), and *GmEIL1d* (c) in the soybean cultivars Suinong 10 (*Phytophthora sojae*‐resistant) and Dongnong 50 (*P. sojae*‐susceptible) following *P. sojae* infection. Samples were collected from 14‐day‐old plants at 0, 9, 12, 24, 48, and 72 h after *P. sojae* infection. Relative expression levels of *GmEIL1b*, *GmEIL1c*, and *GmEIL1d* were compared at each time point with those in negative control plants treated with sterile water. *GmEF1b* (NM_001248778) was used as the internal control to normalize all data. Statistical analyses of (a–c) were performed using three biological replicates, each with three technical replicates. Data were analysed using Student’s *t* test (*p < 0.05, **p < 0.01). Error bars indicate the standard errors of the means.

**FIGURE S5.** Relative expression of *GmEIL1* and three homologous genes in roots of transgenic plants. Relative transcript abundance of *GmEIL1* (a), *GmEIL1b* (b), *GmEIL1*c (c), and *GmEIL1d* (d) in *GmEIL1* transgenic and wild‐type (WT) Dongnong 50 plants. *GmEF1b* was used as the internal control to normalize all data. The experiment was performed using three biological replicates, each with three technical replicates. Data were analysed using Student’s *t* test (*p < 0.05, **p < 0.01). Error bars indicate the standard errors of the means.

**FIGURE S6.** Relative expression of *GmEIL1* and three homologous genes in cotyledons of transgenic plants. Relative transcript abundance of *GmEIL1* (a), *GmEIL1b* (b), *GmEIL1c* (c), and *GmEIL1d* (d) in *GmEIL1* transgenic and wild‐type (WT) Dongnong 50 plants. *GmEF1b* was used as the internal control to normalize all data. The experiment was performed using three biological replicates, each with three technical replicates. Data were analysed using Student’s *t* test (*p < 0.05, **p < 0.01). Error bars indicate the standard errors of the means.

**FIGURE S7.** Transcriptomic analysis of gene expression profiles in response to *GmEIL1* overexpression (OE). (a) Volcano plots of differential expressed genes (DEGs) in empty vector (EV)‐containing control hairy roots and *GmEIL1*‐OE transgenic hairy roots after the RNA‐seq analysis. (b) Gene ontology functional classification of the DEGs, which were placed into the three main categories: biological process, cellular component, and molecular function.

**FIGURE S8.** Chromatin immunoprecipitation‐quantitative PCR analysis of GmEIL1 binding to promoters of other differentially expressed genes. Precipitated chromatin fragments were analysed by quantitative PCR using a primer targeted upstream of *GmRPM* (a,b), *GmRAP2.6* (c,d), *GmABP* (e), and *GmRFL* (f,g). P1 and P2 represent two EBS sequences in the promoters of differentially expressed genes. One‐tenth of the input chromatin (without antibody precipitation) was used as a control. Data represent the means of three biological replicates, each with three technical replicates, and were analysed using Student’s *t* test (*p < 0.05, **p < 0.01). Error bars indicate standard errors of the means.

**FIGURE S9.** Effects of exogenous application of ethylene on soybean resistance to *Phytophthora sojae*. Cotyledons of *GmEIL1* transgenic and wild‐type (WT; Dongnong 50) plants were inoculated with *P. sojae* for 5 days along with exogenous ethylene treatment. Quantitative PCR analysis of the relative biomass of *P. sojae* in *GmEIL1* transgenic and WT cotyledons based on *P. sojae TEF1* transcript levels. *GmEF1b* was used as the internal control to normalize all data. Statistical analyses were performed using three biological replicates, each with three technical replicates. Statistical significance was determined using Student’s *t* test (*p < 0.05, **p < 0.01). Error bars indicate the standard errors of the means.

**FIGURE S10.** Effect of GmEIL1 on the transcriptional activity of ethylene biosynthesis‐related genes. (a–c) Representative images of a dual luciferase assay in *Nicotiana benthamiana* leaves. The results show that GmEIL1 activated the expression of *GmACS09* (b) by binding to its promoter but did not bind to the promoter of *GmACS02* (a) and *GmACO3* (c). (d–f) Detection of LUC/REN activity to verify that GmEIL1 activated the transcription of *GmACS09* (e) but not *GmACS02* (d) and *GmACO3* (f). The combination of the reporter construct (*GmACS02*‐LUC or *GmACS09*‐LUC or *GmACO3*‐LUC) and the emty vector construct (EV) was used as the control. Data represent the means of three biological replicates, each with three technical replicates. Data were analysed using Student’s *t* test (*p < 0.05, **p < 0.01). Error bars indicate standard errors of the means.

**FIGURE S11.** Expression patterns of *GmEIL1* following ethylene treatment of the *GmEIL1*‐OE and *GmEIL1*‐RNAi transgenic soybean plants. Statistical analyses were performed using three biological replicates, each with three technical replicates. Data were analysed using Student’s *t* test (*p < 0.05, **p < 0.01). Error bars indicate the standard errors of the means.

**FIGURE S12.** Aminocyclopropane‐1‐carboxylic acid (ACC) contents in roots and cotyledons of *GmEIL1*‐OE, *GmEIL1*‐RNAi, and wild‐type (WT, Dongnong 50) soybean plants infected with *Phytophthora sojae*. Statistical analyses were performed using three biological replicates, each with three technical replicates. Data were analysed using Student’s *t* test (*p < 0.05, **p < 0.01). Error bars indicate the standard errors of the means.

**FIGURE S13.** Expression patterns of *GmACS02* (a), *GmACS09* (b), and *GmACO3* (c). in response to *Phytophthora sojae* infection of resistant cultivar Suinong 10 versus susceptible cultivar Dongnong 50. Samples were collected at 0, 9, 12, 24, 48, and 72 h after *P. sojae* infection. *GmEF1b* was used as the internal control. Statistical analyses in (a–c) were performed using three biological replicates, each with three technical replicates. Data were analysed using Student’s *t* test (*p < 0.05, **p < 0.01). Error bars indicate the standard errors of the means.

**FIGURE S14.** Effect of GmEIL1 on the transcriptional activity of ethylene pathway‐related genes. (a–g) Representative images of a dual luciferase assay in *Nicotiana benthamiana* leaves showing that GmEIL1 did not bind to its promoter to activate the expression of ethylene pathway‐related genes. (h–n) Detection of LUC/REN activity to verify that GmEIL1 did not activate the transcription of ethylene pathway‐related genes. The combination of the reporter construct (ethylene pathway‐related gene fused with luciferase [LUC]) and the empty vector construct (EV) was used as the control. Data represent the means of three biological replicates, each with three technical replicates, and were analysed using Student’s *t* test (*p < 0.05, **p < 0.01). Error bars indicate standard errors of the means.

**FIGURE S15.** Expression patterns of genes related to ethylene pathway and disease resistance in response to *Phytophthora sojae* infection of resistant cultivar Suinong 10 versus susceptible cultivar Dongnong 50. Samples were collected at 0, 9, 12, 24, 48, and 72 h after *P. sojae* infection. *GmEF1b* was used as the internal control. The statistical analyses in (a–i) were performed using three biological replicates, each with three technical replicates. Data were analysed using Student’s *t* test (*p < 0.05, **p < 0.01). Error bars indicate the standard errors of the means.

**FIGURE S16.** Relative expression levels of *GmPR1* in *GmEIL1* transgenic plants. Relative transcript level of *GmPR1* in *GmEIL1* transgenic, Suinong 10 (SN10), and Dongnong 50 (DN50 WT) soybean plants. *GmEF1b* was used as the internal control to normalize all data. The statistical analyses were performed using three biological replicates, each with three technical replicates. Data were analysed using Student’s *t* test (*p < 0.05, **p < 0.01). Error bars indicate the standard errors of the means.

**FIGURE S17.** Expression patterns of *GmPR1* in response to ethylene treatment of *Phytophthora sojae‐*resistant and ‐susceptible soybean cultivars. Relative expression of *GmPR1* in 14‐day‐old plants of Suinong 10 (resistant) and Dongnong 50 (susceptible) in response to exogenous ethylene treatment for 0, 1, 3, 6, 9, 12, and 24 h. Relative expression levels of GmEIL1 were compared with those in negative control plants (plants treated with sterile water) at the same time point and those at each time point were compared to the corresponding 0 hours. *GmEF1b* was used as the internal control to normalize all data. Statistical analyses were performed using three biological replicates, each with three technical replicates. Data were analysed using Student’s *t* test (*p < 0.05, **p < 0.01). Error bars indicate the standard errors of the means.

**TABLE S1.** Oligonucleotide primers used in this study.

## Data Availability

The data that support the findings of this study are available from the corresponding author upon reasonable request.
