# Supplementary figures and images for: The EIN3 transcription factor GmEIL1 improves soybean resistance to Phytophthora sojae
Source: Mol Plant Pathol. 2024 Apr 15;25(4):e13452. doi: 10.1111/mpp.13452 (PMC11018115; doi:10.1111/mpp.13452)

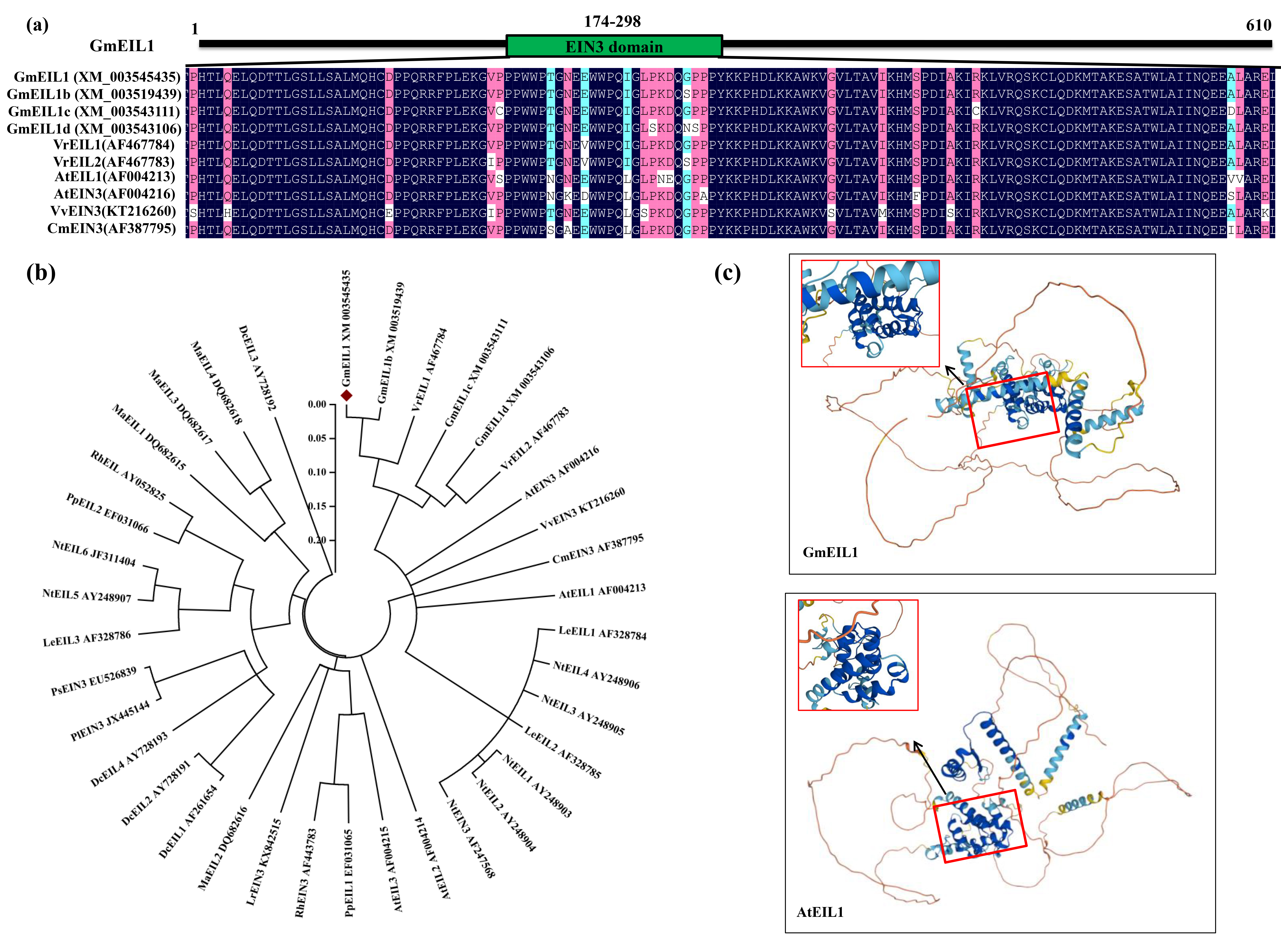

Supplement: Supplementary file 1 — FIGURE S1. Sequence comparison between GmEIL1 and related EIN3/EILs family proteins. (a) Conserved EIN3 domain sequence at amino acids 174–298 of GmEIL1, EIN3, and EIL proteins. (b) Phylogenetic tree reconstructed using soybean GmEIL1, EIN3, and EIL amino acid sequences from various plant species. Amino acid sequences of 37 dirigent domains were analysed using MEGA 5.1. The source species for the EIN3 and EIL1 proteins are as follows: At, Arabidopsis thaliana; Gm, Glycine max; Pp, Podophyllum peltatum; Ps, Pisum sativum; Cm, Cucumis melo; Vr, Vigna radiata; Nt, Nicotiana tabacum; Vv, Vitis vinifera; Le, Solanum lycopersicum; Dc, Dianthus caryophyllus; Rh, Rosa hybrida; Lr, Lilium regale; Ma, Musa acuminata; Pl, Paeonia lactiflora. (c) Three‐dimensional structure of GmEIL1 and AtEIL1, with the EIN3 domain labelled. [file MPP-25-e13452-s009.jpg]

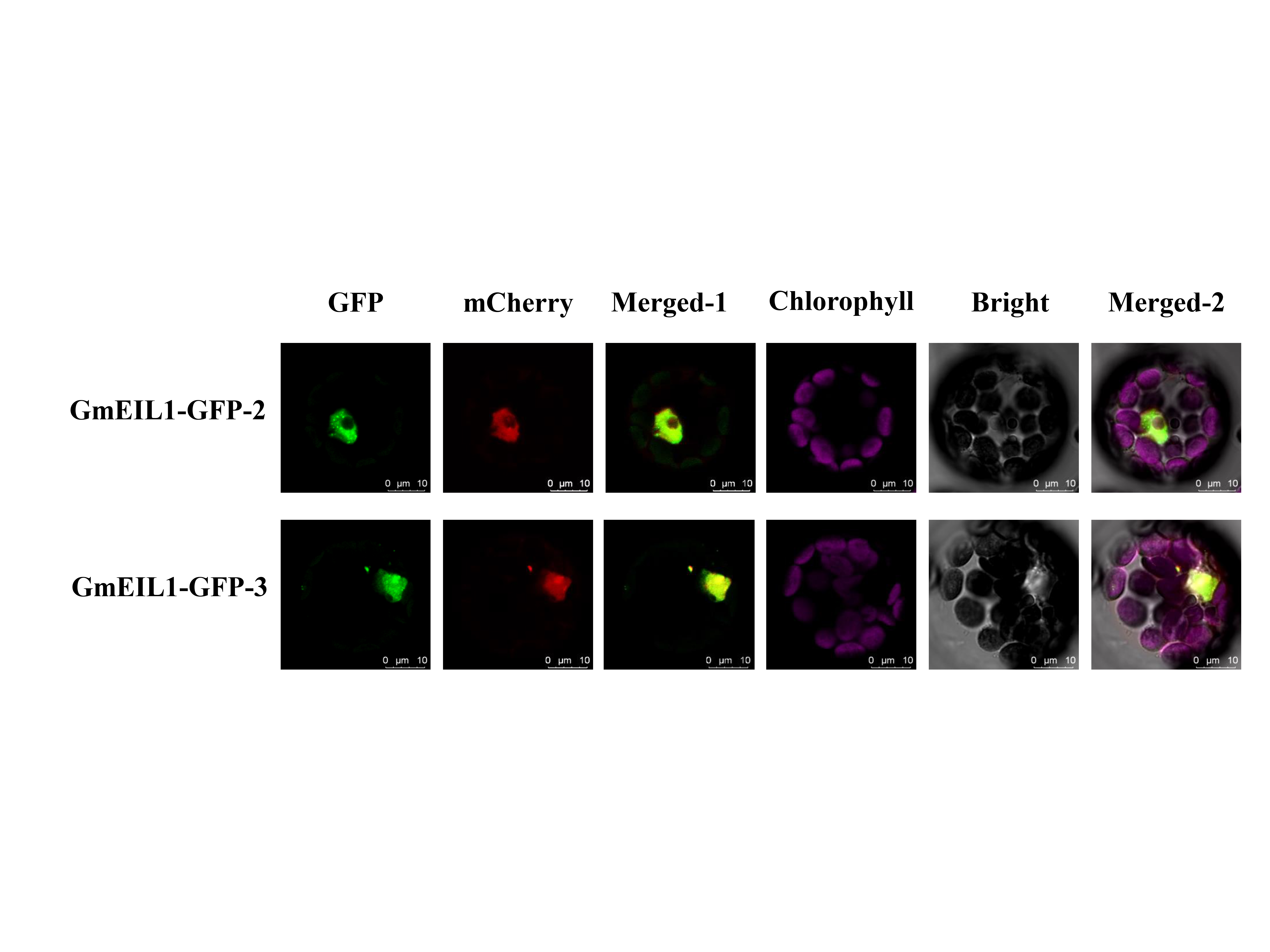

Supplement: Supplementary file 2 — FIGURE S2. Subcellular localization of GmEIL1. GmEIL1‐GFP and H2B‐mCherry fusion plasmids (or 35S:GFP and the H2B marker gene for the nucleus) were cotransformed into Arabidopsis protoplasts using a polyethylene glycol‐mediated method. Bright‐field images, GFP fluorescence (green), chlorophyll autofluorescence (purple), mCherry fluorescence (magenta), and the merged images are shown. Merged‐1 is a merged image of the nucleus marker control (H2B‐mCherry) and GFP channels, Merged‐2 is a merged image of all channels. Size bars indicate 10 μm. The fluorescence images indicated that GmEIL1 localizes in the nucleus. [file MPP-25-e13452-s002.jpg]

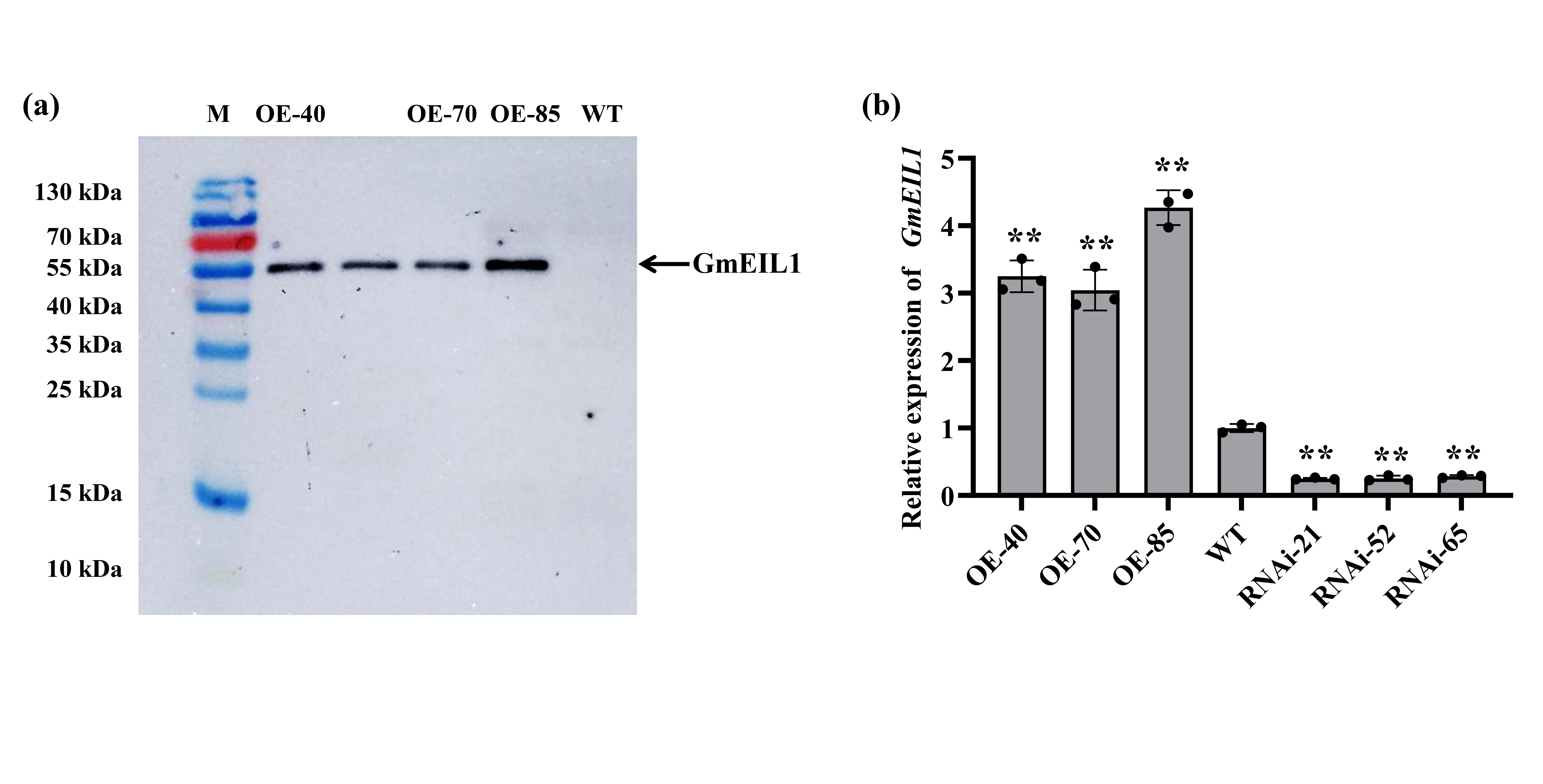

Supplement: Supplementary file 3 — FIGURE S3. Verification of GmEIL1 overexpression and silencing in transgenic soybean plants. (a) Immunoblot analysis of GmEIL1 expression in three overexpressing transgenic soybean lines (OE‐40, OE‐70, and OE‐85). (b) Reverse transcription‐quantitative PCR analysis of the relative expression of GmEIL1 in GmEIL1‐OE, GmEIL1‐RNAi, and wild‐type (WT: Dongnong 50) soybean plants. [file MPP-25-e13452-s006.jpg]

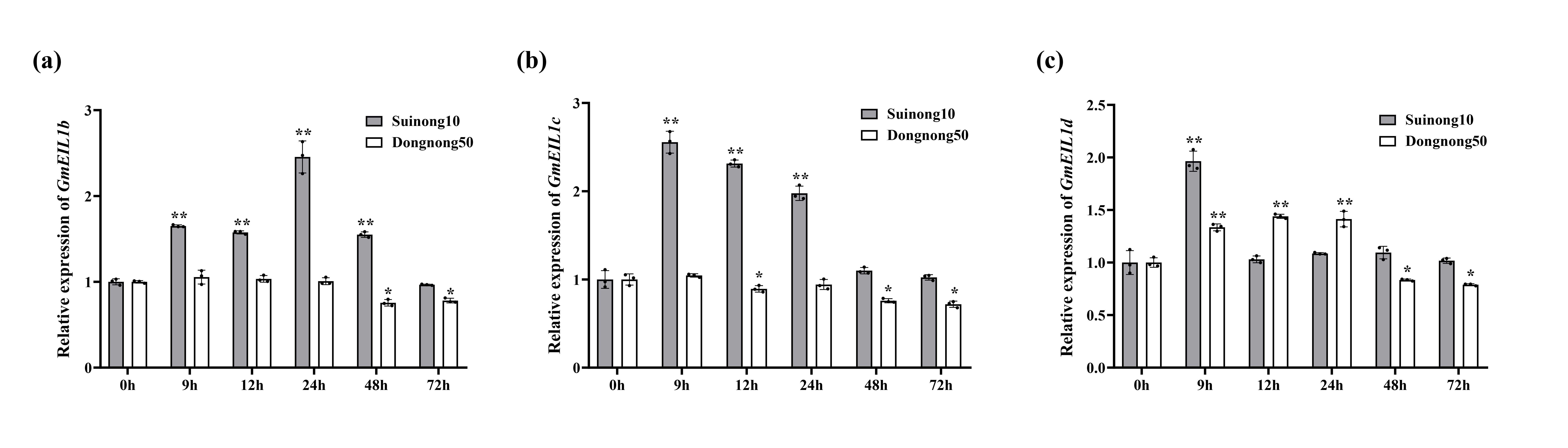

Supplement: Supplementary file 4 — FIGURE S4. Expression patterns of GmEIL1b, GmEIL1c, and GmEIL1d in resistant cultivar Suinong 10 versus susceptible cultivars Dongnong 50. Relative expression of GmEIL1b (a), GmEIL1c (b), and GmEIL1d (c) in the soybean cultivars Suinong 10 (Phytophthora sojae‐resistant) and Dongnong 50 (P. sojae‐susceptible) following P. sojae infection. Samples were collected from 14‐day‐old plants at 0, 9, 12, 24, 48, and 72 h after P. sojae infection. Relative expression levels of GmEIL1b, GmEIL1c, and GmEIL1d were compared at each time point with those in negative control plants treated with sterile water. GmEF1b (NM_001248778) was used as the internal control to normalize all data. Statistical analyses of (a–c) were performed using three biological replicates, each with three technical replicates. Data were analysed using Student’s t test (*p < 0.05, **p < 0.01). Error bars indicate the standard errors of the means. [file MPP-25-e13452-s012.jpg]

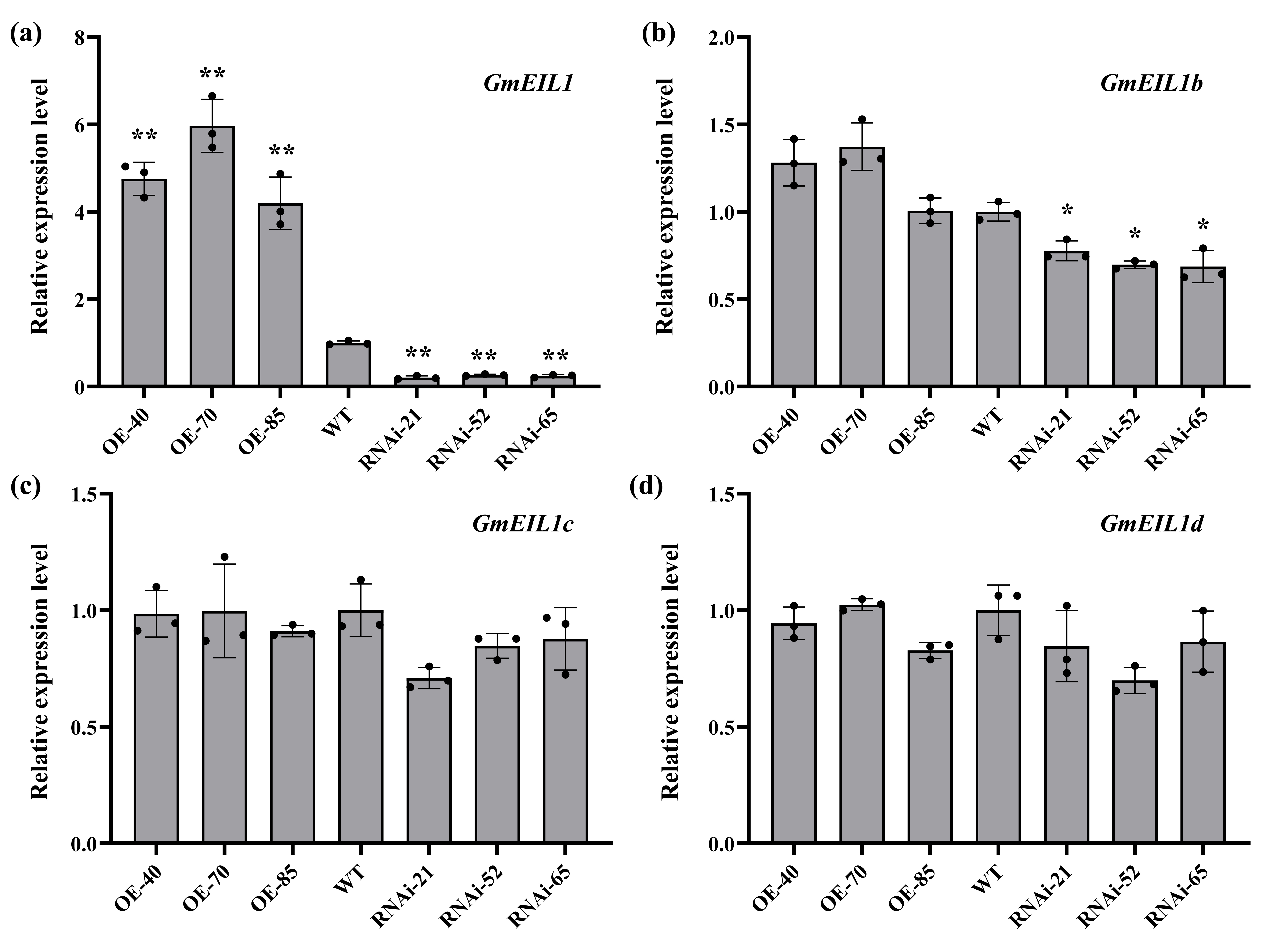

Supplement: Supplementary file 5 — FIGURE S5. Relative expression of GmEIL1 and three homologous genes in roots of transgenic plants. Relative transcript abundance of GmEIL1 (a), GmEIL1b (b), GmEIL1c (c), and GmEIL1d (d) in GmEIL1 transgenic and wild‐type (WT) Dongnong 50 plants. GmEF1b was used as the internal control to normalize all data. The experiment was performed using three biological replicates, each with three technical replicates. Data were analysed using Student’s t test (*p < 0.05, **p < 0.01). Error bars indicate the standard errors of the means. [file MPP-25-e13452-s005.jpg]

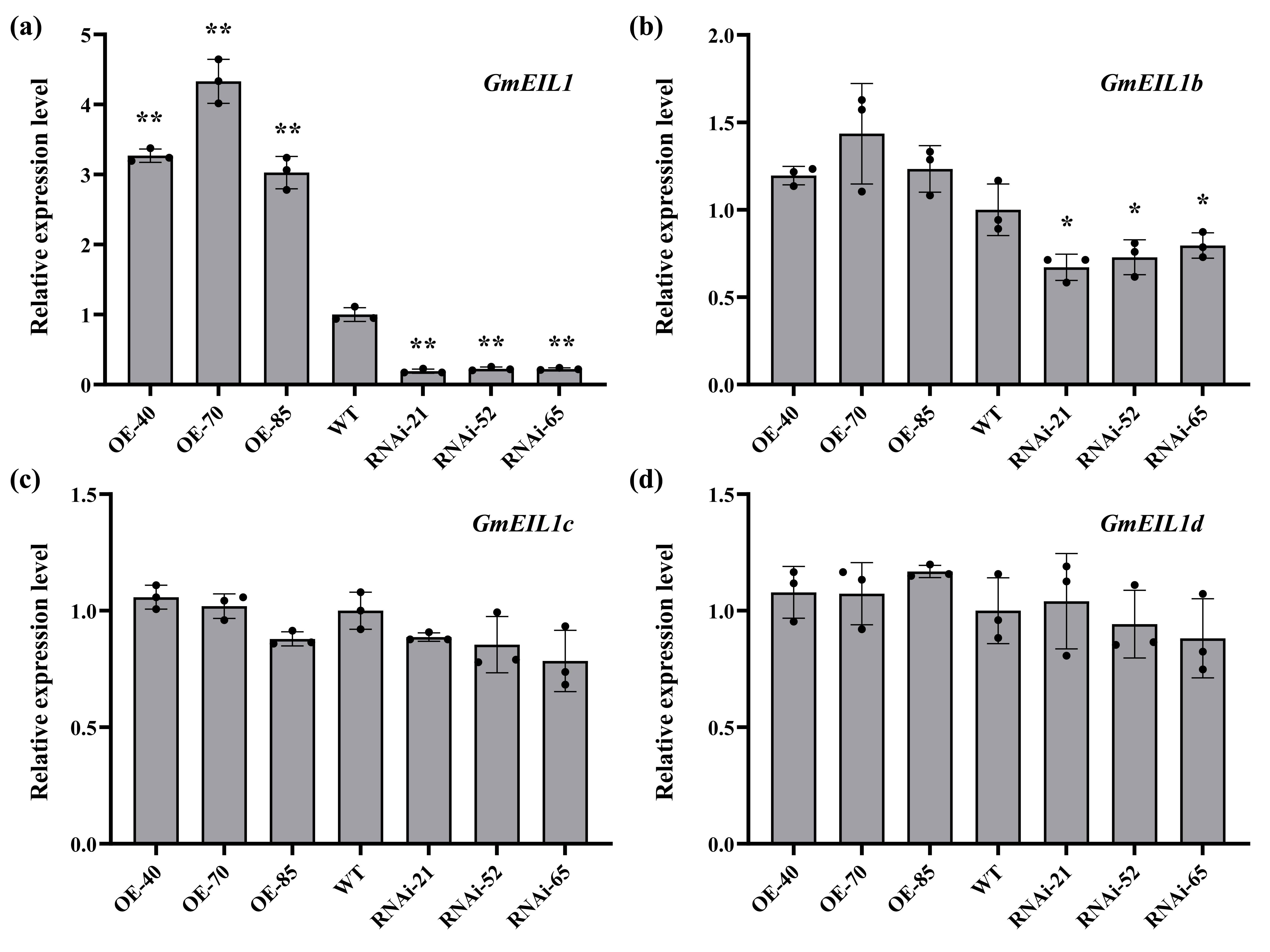

Supplement: Supplementary file 6 — FIGURE S6. Relative expression of GmEIL1 and three homologous genes in cotyledons of transgenic plants. Relative transcript abundance of GmEIL1 (a), GmEIL1b (b), GmEIL1c (c), and GmEIL1d (d) in GmEIL1 transgenic and wild‐type (WT) Dongnong 50 plants. GmEF1b was used as the internal control to normalize all data. The experiment was performed using three biological replicates, each with three technical replicates. Data were analysed using Student’s t test (*p < 0.05, **p < 0.01). Error bars indicate the standard errors of the means. [file MPP-25-e13452-s016.jpg]

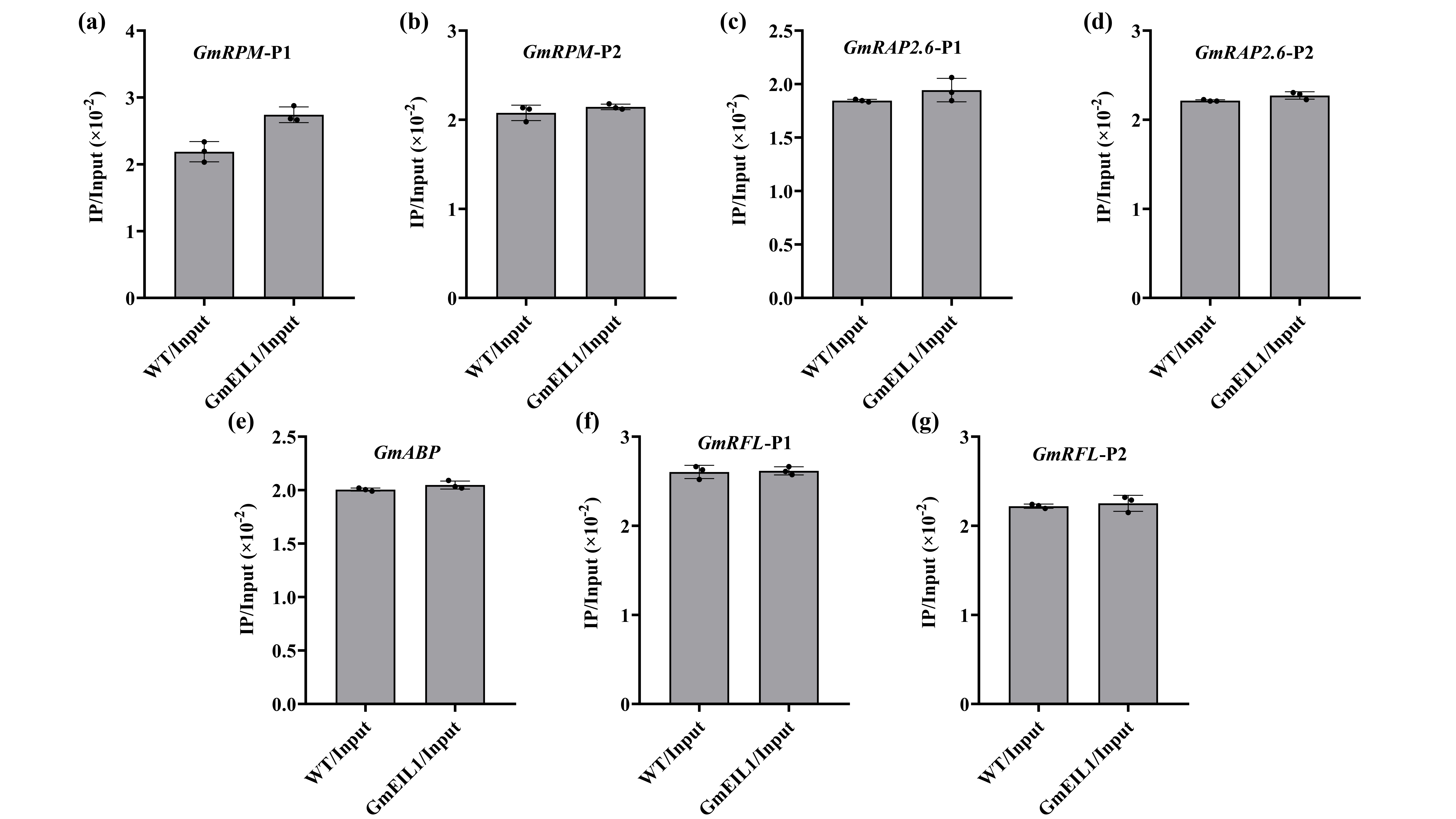

Supplement: Supplementary file 8 — FIGURE S8. Chromatin immunoprecipitation‐quantitative PCR analysis of GmEIL1 binding to promoters of other differentially expressed genes. Precipitated chromatin fragments were analysed by quantitative PCR using a primer targeted upstream of GmRPM (a,b), GmRAP2.6 (c,d), GmABP (e), and GmRFL (f,g). P1 and P2 represent two EBS sequences in the promoters of differentially expressed genes. One‐tenth of the input chromatin (without antibody precipitation) was used as a control. Data represent the means of three biological replicates, each with three technical replicates, and were analysed using Student’s t test (*p < 0.05, **p < 0.01). Error bars indicate standard errors of the means. [file MPP-25-e13452-s010.jpg]

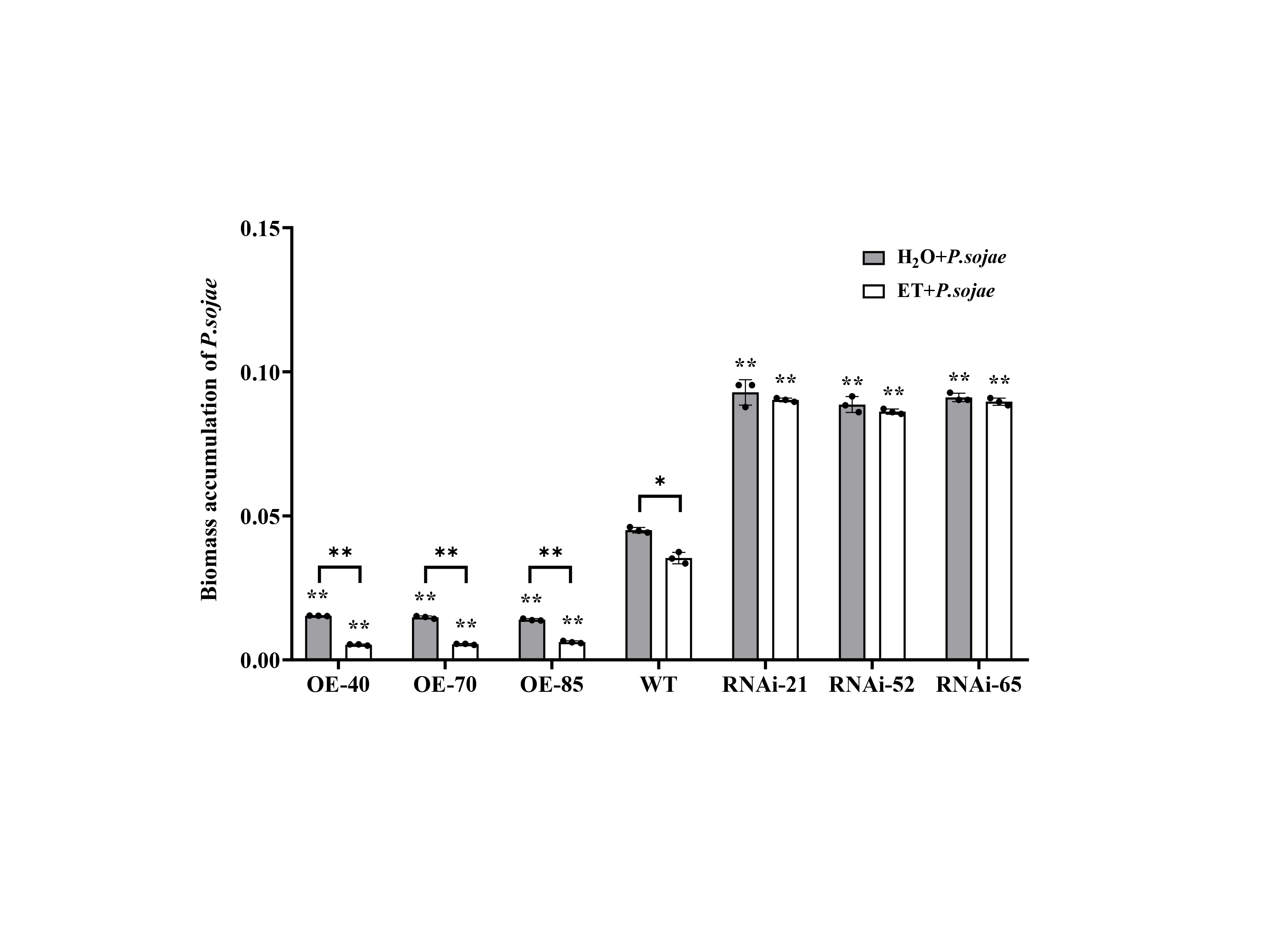

Supplement: Supplementary file 9 — FIGURE S9. Effects of exogenous application of ethylene on soybean resistance to Phytophthora sojae. Cotyledons of GmEIL1 transgenic and wild‐type (WT; Dongnong 50) plants were inoculated with P. sojae for 5 days along with exogenous ethylene treatment. Quantitative PCR analysis of the relative biomass of P. sojae in GmEIL1 transgenic and WT cotyledons based on P. sojae TEF1 transcript levels. GmEF1b was used as the internal control to normalize all data. Statistical analyses were performed using three biological replicates, each with three technical replicates. Statistical significance was determined using Student’s t test (*p < 0.05, **p < 0.01). Error bars indicate the standard errors of the means. [file MPP-25-e13452-s001.jpg]

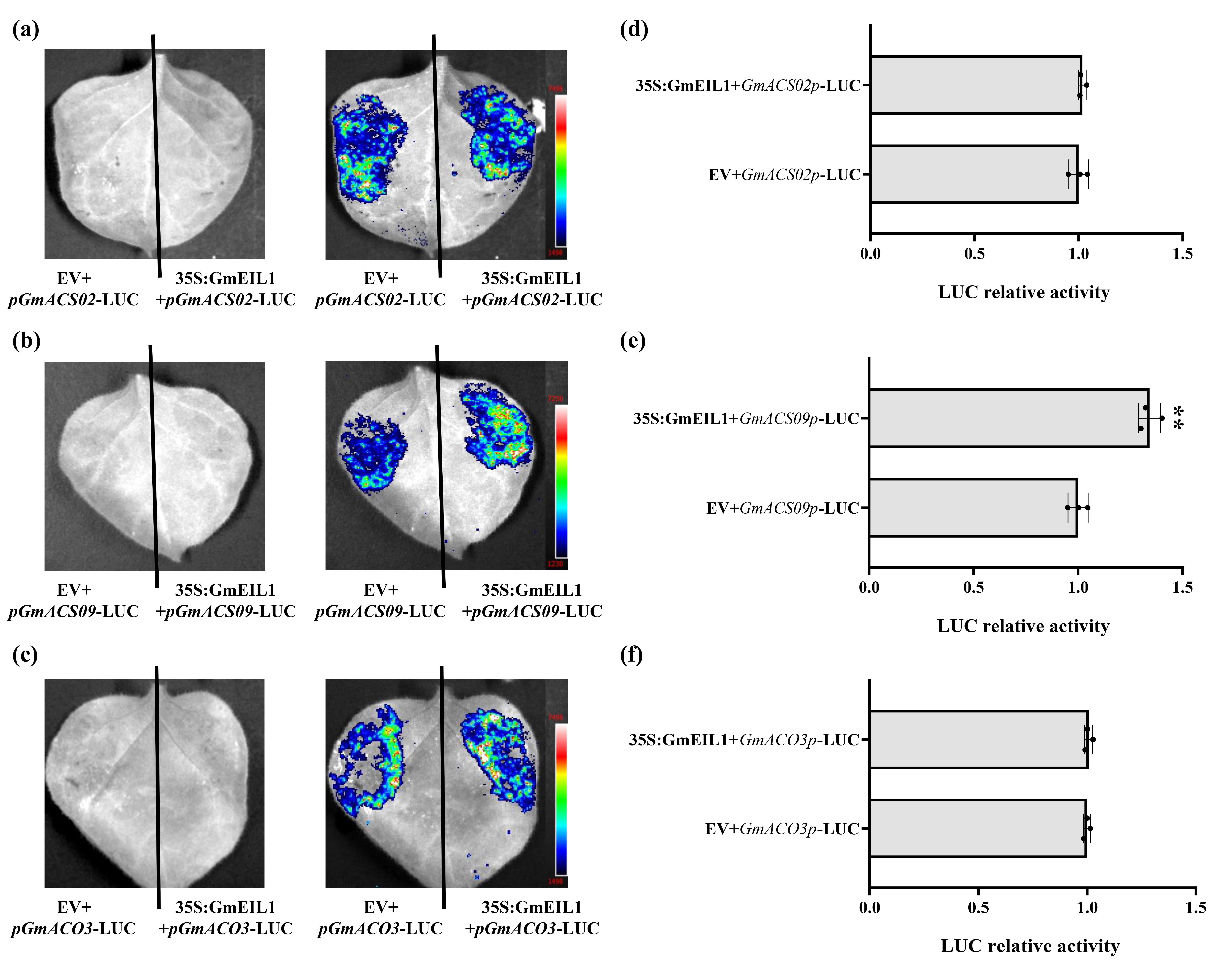

Supplement: Supplementary file 10 — FIGURE S10. Effect of GmEIL1 on the transcriptional activity of ethylene biosynthesis‐related genes. (a–c) Representative images of a dual luciferase assay in Nicotiana benthamiana leaves. The results show that GmEIL1 activated the expression of GmACS09 (b) by binding to its promoter but did not bind to the promoter of GmACS02 (a) and GmACO3 (c). (d–f) Detection of LUC/REN activity to verify that GmEIL1 activated the transcription of GmACS09 (e) but not GmACS02 (d) and GmACO3 (f). The combination of the reporter construct (GmACS02‐LUC or GmACS09‐LUC or GmACO3‐LUC) and the emty vector construct (EV) was used as the control. Data represent the means of three biological replicates, each with three technical replicates. Data were analysed using Student’s t test (*p < 0.05, **p < 0.01). Error bars indicate standard errors of the means. [file MPP-25-e13452-s011.jpg]

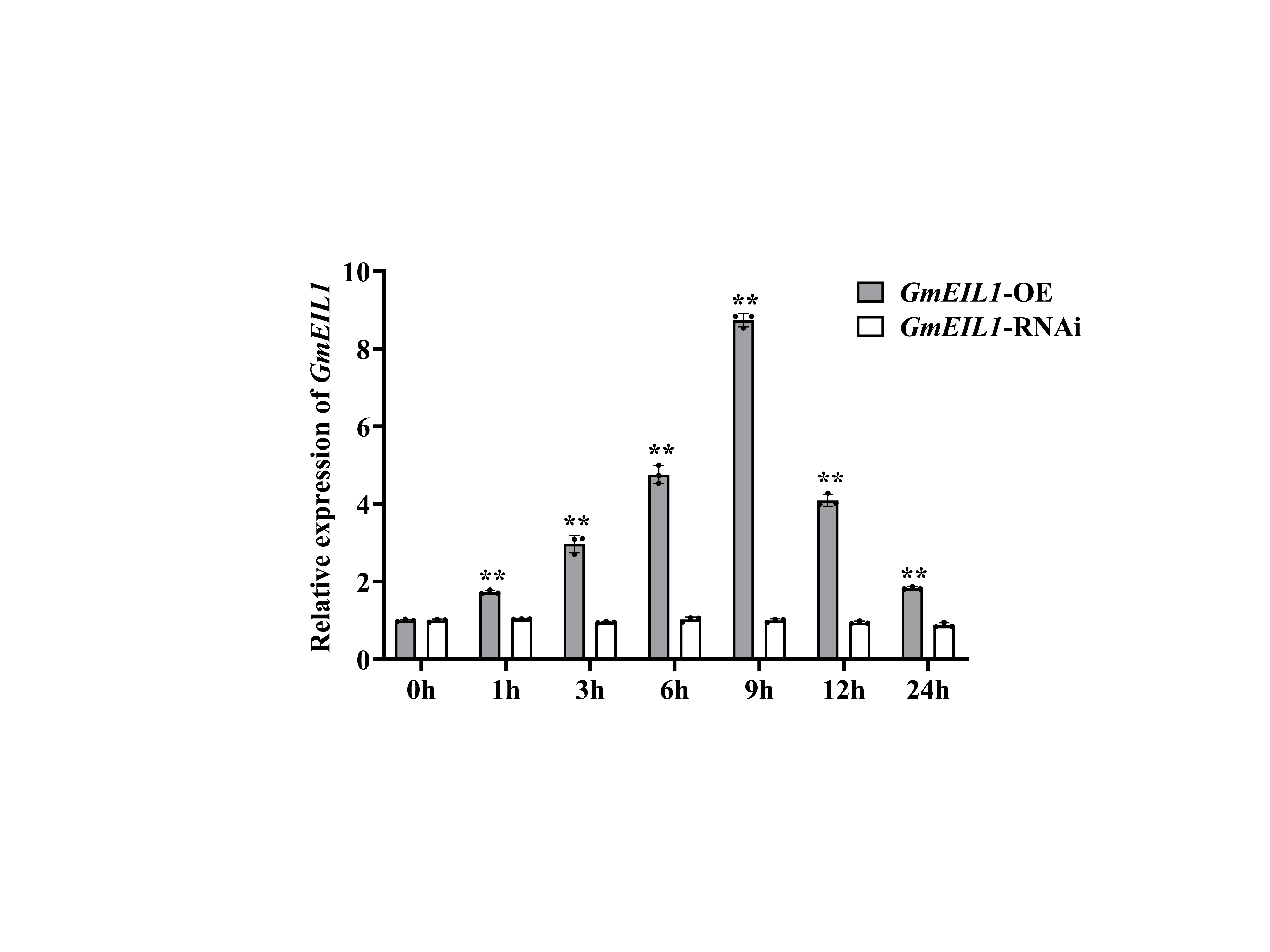

Supplement: Supplementary file 11 — FIGURE S11. Expression patterns of GmEIL1 following ethylene treatment of the GmEIL1‐OE and GmEIL1‐RNAi transgenic soybean plants. Statistical analyses were performed using three biological replicates, each with three technical replicates. Data were analysed using Student’s t test (*p < 0.05, **p < 0.01). Error bars indicate the standard errors of the means. [file MPP-25-e13452-s015.jpg]

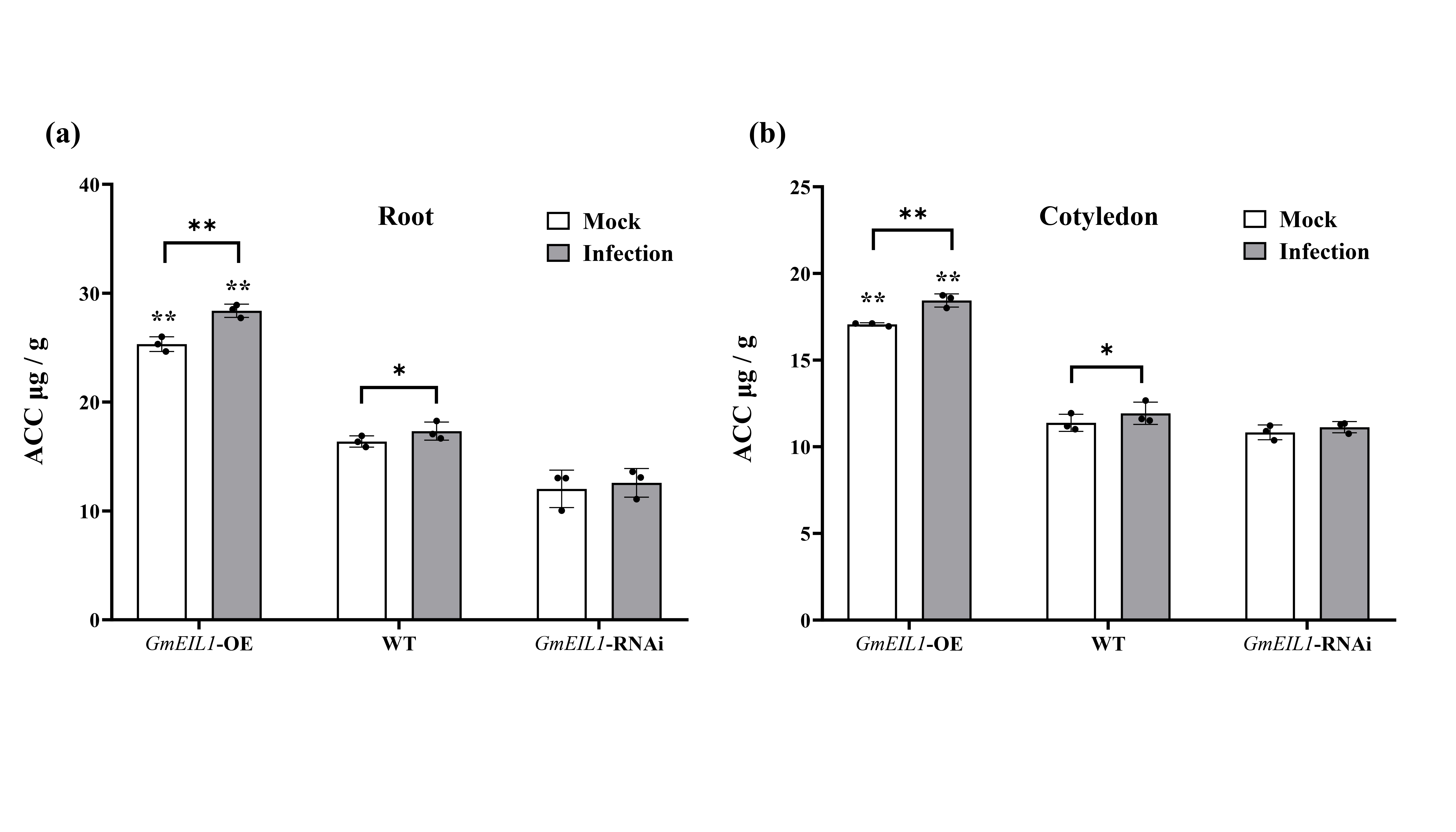

Supplement: Supplementary file 12 — FIGURE S12. Aminocyclopropane‐1‐carboxylic acid (ACC) contents in roots and cotyledons of GmEIL1‐OE, GmEIL1‐RNAi, and wild‐type (WT, Dongnong 50) soybean plants infected with Phytophthora sojae. Statistical analyses were performed using three biological replicates, each with three technical replicates. Data were analysed using Student’s t test (*p < 0.05, **p < 0.01). Error bars indicate the standard errors of the means. [file MPP-25-e13452-s007.jpg]

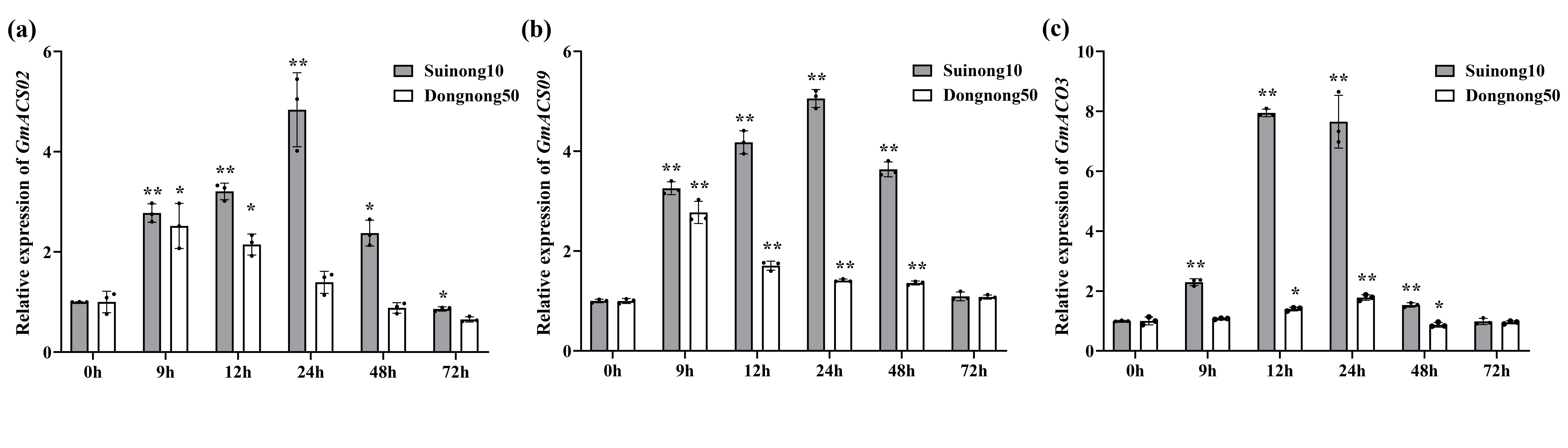

Supplement: Supplementary file 13 — FIGURE S13. Expression patterns of GmACS02 (a), GmACS09 (b), and GmACO3 (c). in response to Phytophthora sojae infection of resistant cultivar Suinong 10 versus susceptible cultivar Dongnong 50. Samples were collected at 0, 9, 12, 24, 48, and 72 h after P. sojae infection. GmEF1b was used as the internal control. Statistical analyses in (a–c) were performed using three biological replicates, each with three technical replicates. Data were analysed using Student’s t test (*p < 0.05, **p < 0.01). Error bars indicate the standard errors of the means. [file MPP-25-e13452-s017.jpg]

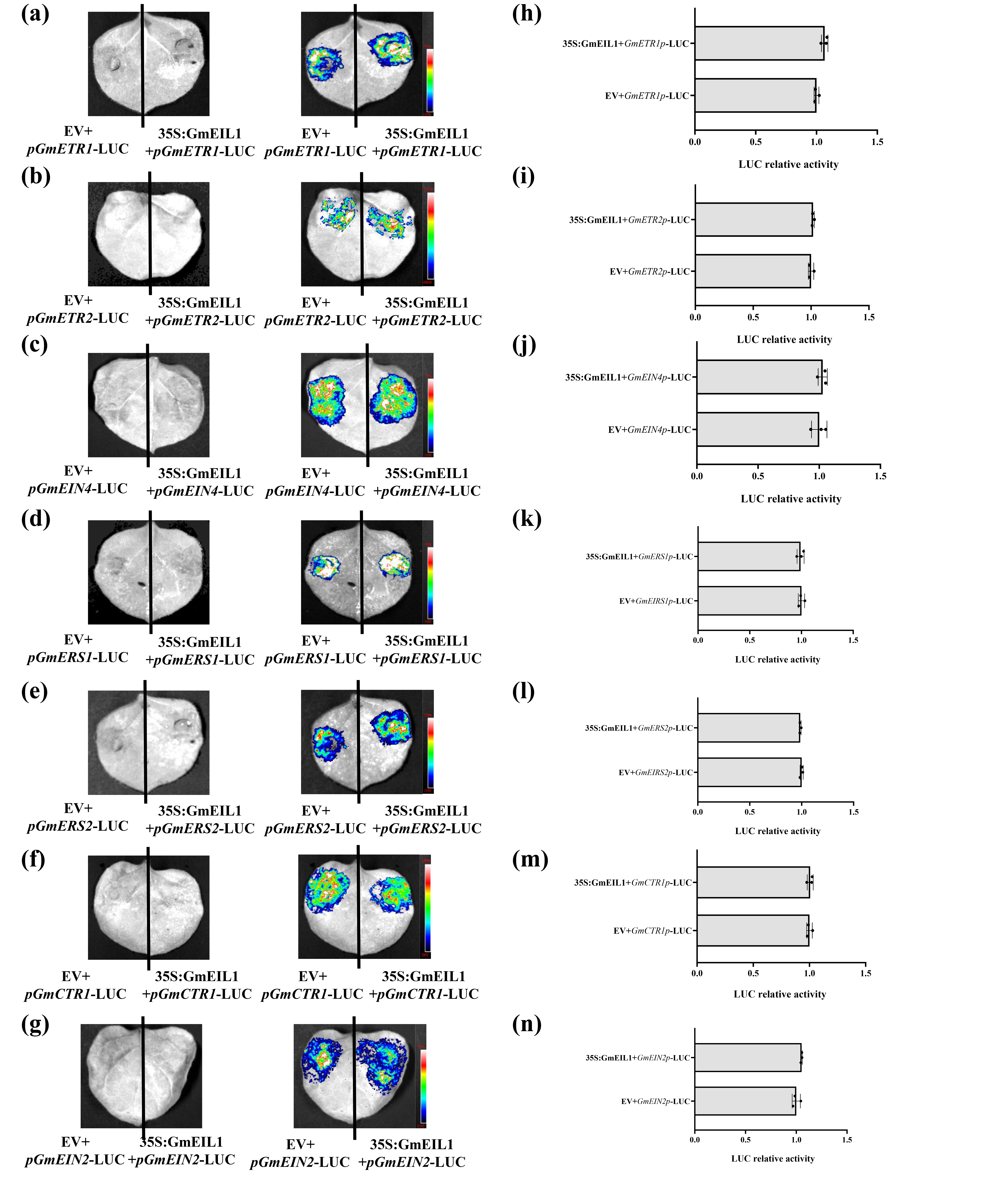

Supplement: Supplementary file 14 — FIGURE S14. Effect of GmEIL1 on the transcriptional activity of ethylene pathway‐related genes. (a–g) Representative images of a dual luciferase assay in Nicotiana benthamiana leaves showing that GmEIL1 did not bind to its promoter to activate the expression of ethylene pathway‐related genes. (h–n) Detection of LUC/REN activity to verify that GmEIL1 did not activate the transcription of ethylene pathway‐related genes. The combination of the reporter construct (ethylene pathway‐related gene fused with luciferase [LUC]) and the empty vector construct (EV) was used as the control. Data represent the means of three biological replicates, each with three technical replicates, and were analysed using Student’s t test (*p < 0.05, **p < 0.01). Error bars indicate standard errors of the means. [file MPP-25-e13452-s003.jpg]

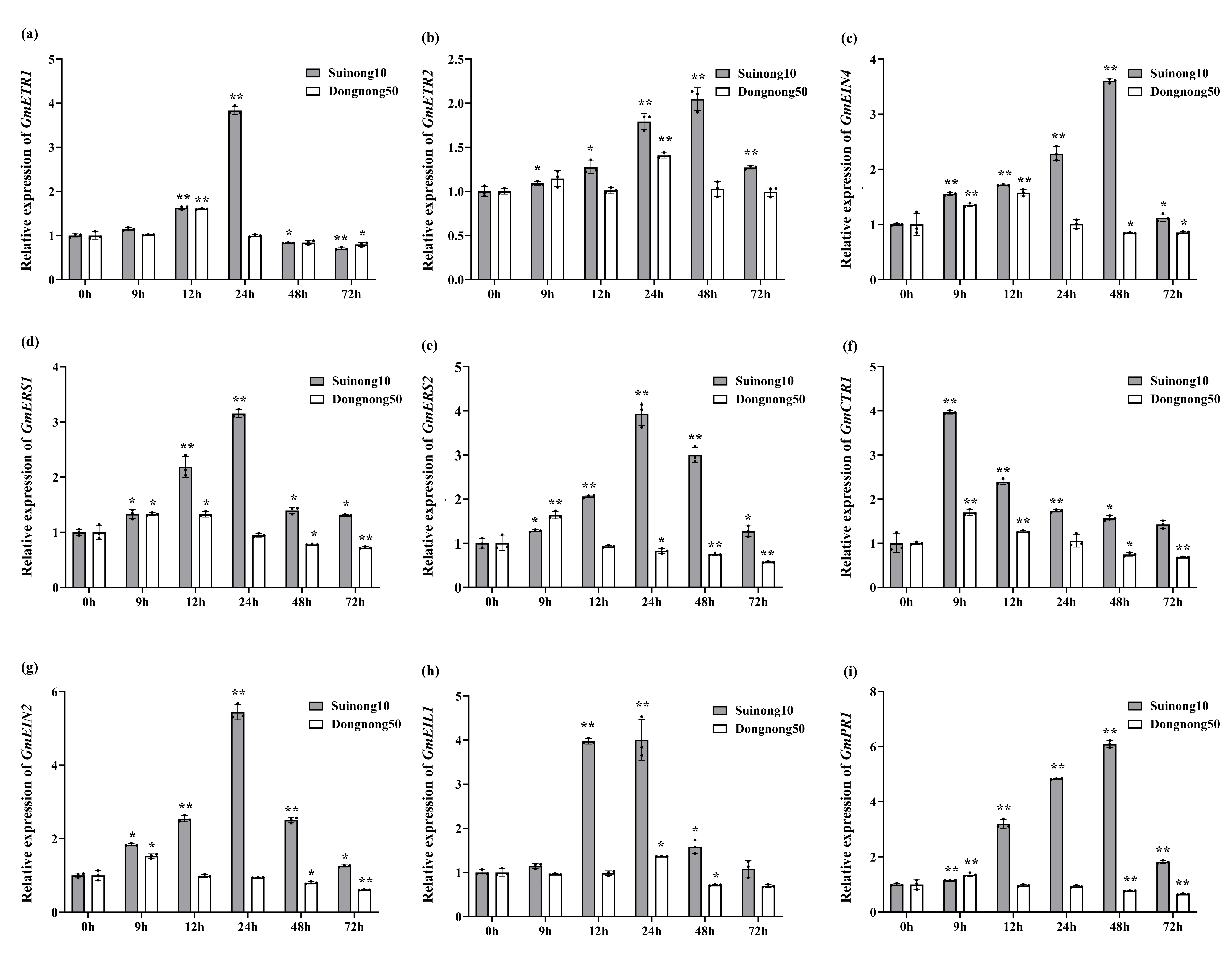

Supplement: Supplementary file 15 — FIGURE S15. Expression patterns of genes related to ethylene pathway and disease resistance in response to Phytophthora sojae infection of resistant cultivar Suinong 10 versus susceptible cultivar Dongnong 50. Samples were collected at 0, 9, 12, 24, 48, and 72 h after P. sojae infection. GmEF1b was used as the internal control. The statistical analyses in (a–i) were performed using three biological replicates, each with three technical replicates. Data were analysed using Student’s t test (*p < 0.05, **p < 0.01). Error bars indicate the standard errors of the means. [file MPP-25-e13452-s018.jpg]

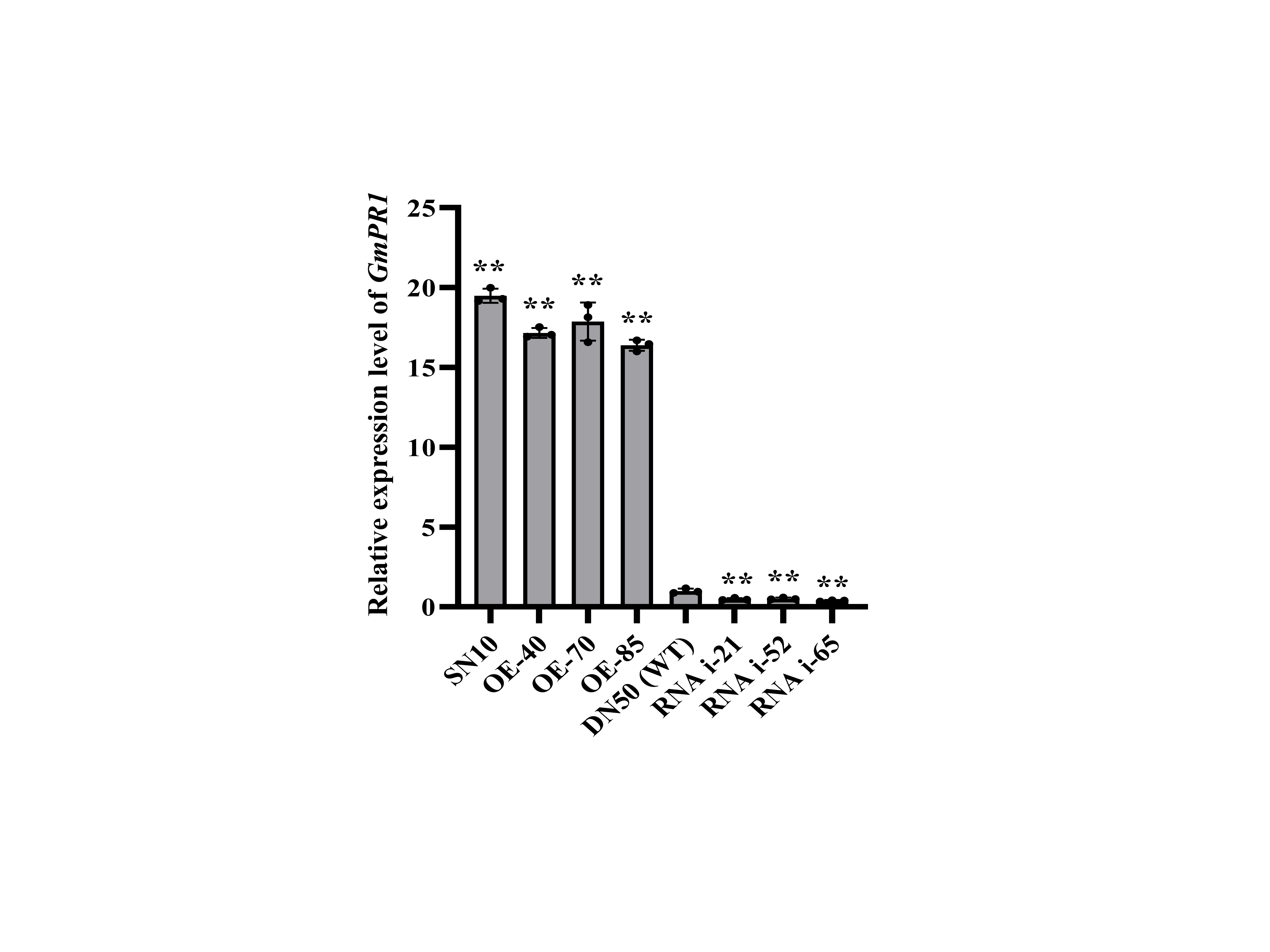

Supplement: Supplementary file 16 — FIGURE S16. Relative expression levels of GmPR1 in GmEIL1 transgenic plants. Relative transcript level of GmPR1 in GmEIL1 transgenic, Suinong 10 (SN10), and Dongnong 50 (DN50 WT) soybean plants. GmEF1b was used as the internal control to normalize all data. The statistical analyses were performed using three biological replicates, each with three technical replicates. Data were analysed using Student’s t test (*p < 0.05, **p < 0.01). Error bars indicate the standard errors of the means. [file MPP-25-e13452-s004.jpg]

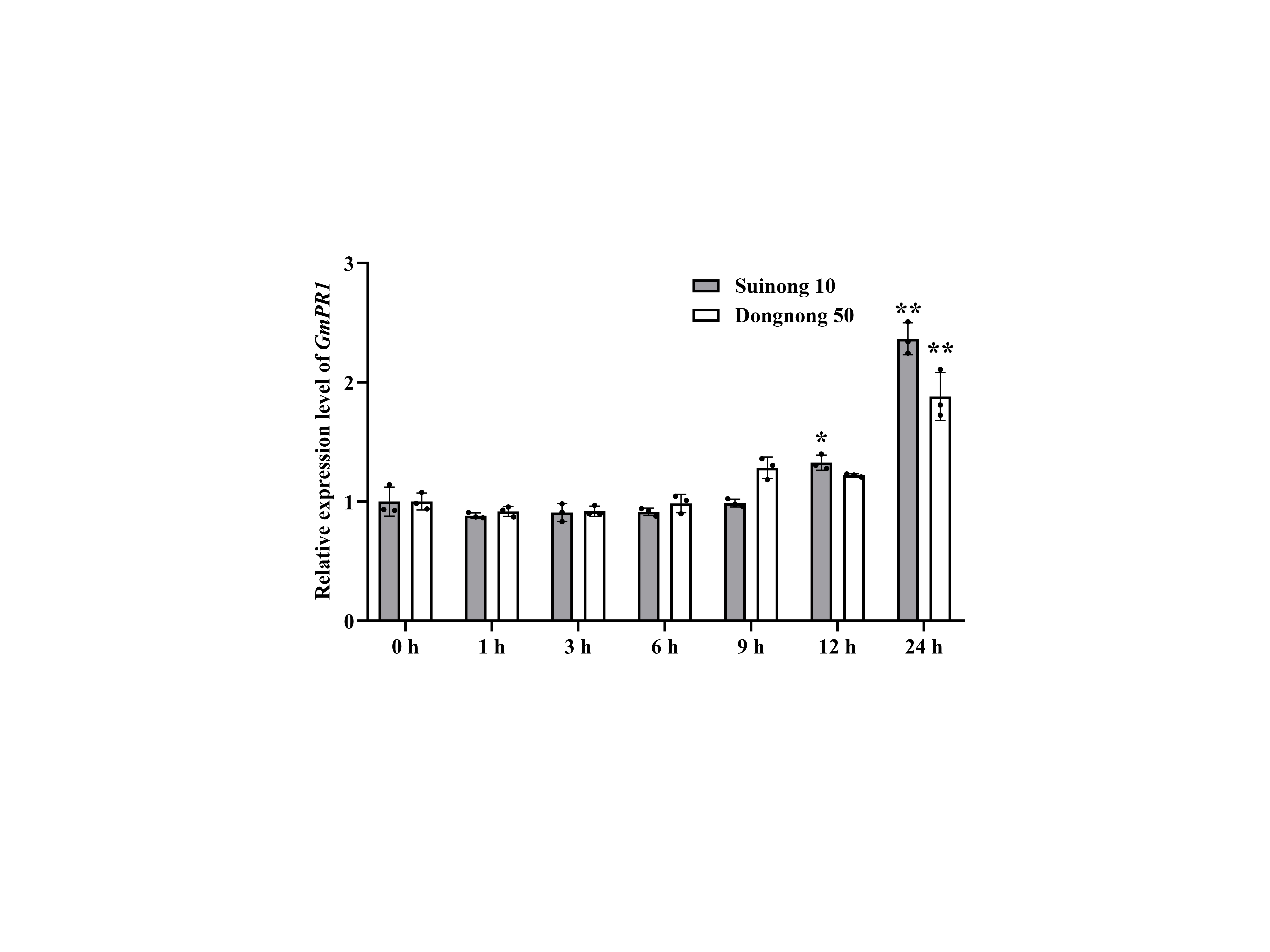

Supplement: Supplementary file 17 — FIGURE S17. Expression patterns of GmPR1 in response to ethylene treatment of Phytophthora sojae‐resistant and ‐susceptible soybean cultivars. Relative expression of GmPR1 in 14‐day‐old plants of Suinong 10 (resistant) and Dongnong 50 (susceptible) in response to exogenous ethylene treatment for 0, 1, 3, 6, 9, 12, and 24 h. Relative expression levels of GmEIL1 were compared with those in negative control plants (plants treated with sterile water) at the same time point and those at each time point were compared to the corresponding 0 hours. GmEF1b was used as the internal control to normalize all data. Statistical analyses were performed using three biological replicates, each with three technical replicates. Data were analysed using Student’s t test (*p < 0.05, **p < 0.01). Error bars indicate the standard errors of the means. [file MPP-25-e13452-s013.jpg]
