# Supplementary material for: The EIN3 transcription factor GmEIL1 improves soybean resistance to Phytophthora sojae
Source: Mol Plant Pathol. 2024 Apr 15;25(4):e13452. doi: 10.1111/mpp.13452 (PMC11018115; doi:10.1111/mpp.13452)
Supplement: Supplementary file 18 — TABLE S1. Oligonucleotide primers used in this study. [file MPP-25-e13452-s008.docx]

**Table S1. Oligonucleotide primers used in this study.**

| **Gene cloning** | *GmEIL1*F | ATGATGATGTTTGAAGATATGGG |
| --- | --- | --- |
|  | *GmEIL1*R | CTGGTACCAAATTGAAACCTCTT |
|  | AD*-GmEIL1*F | GGAATTCATGATGATGTTTGAAGATATGGGAT |
|  | AD*-GmEIL1*R | CGAGCTCACCTCCCAAAGCATAAAATA |
|  | BD*-GmEIL1*F | GGAATTCATGATGATGTTTGAAGATATGGGAT |
|  | BD*-GmEIL1*R | GGTCGACACCTCCCAAAGCATAAAATA |
|  | *GmEIL1-*oF | CAGATCT ATGATGATGTTTGAAGATATGGG |
|  | *GmEIL1-*oR | CCACGTG CTGGTACCAAATTGAAACCTCTT |
|  | *GmEIL1-*rF1 | CCTCGAGCAAACCTTGGGATGGAGA |
|  | *GmEIL1-*rR1 | CCCATGGCTGGCTTAACCTCAGTAGCA |
|  | *GmEIL1-*rF2 | CTCTAGACAAACCTTGGGATGGAGA |
|  | *GmEIL1-*rR2 | CGGATCCCTGGCTTAACCTCAGTAGCA |
|  | *GmERF113-*pF | GCTCGAG GGCAGCATCCCGCAT |
|  | *GmERF113-*pR | GCCATGG GCTCTTAGGTGGAGATTGGTTA |
| **RT-qPCR** | *GmEIL1-*qF | ATGGGATTCTGTGGCGATTT |
|  | *GmEIL1-*qR | GCTCTTGGGATTGCCTTTG |
|  | *GmEF1*bF | CCACTGCTGAAGAAGATGATGATG |
|  | *GmEF1*bR | AAGGACAGAAGACTTGCCACTC |
|  | *GmTEF1*F | TGATCGTGCTGAACCACCC |
|  | *GmTEF1*R | CGAGCGACGGTCCATCTT |
|  | *GmRPM-*qF | CAATCAGTGGTGTCCTGGCTAC |
|  | *GmRPM-*qR | GCTCCAAGACTACGGCAAATC |
|  | *GmERF113-*qF | GATAGCACCCTTTCTTCACCAA |
|  | *GmERF113-*qR | ATGTCTTTTTCTCCCATTTCCT |
|  | *GmRAP2.6-*qF | CCTTCTCCCGTTCTGTCTTCTT |
|  | *GmRAP2.6-*qR | CAACACCTCCACTCGCACC |
|  | *GmRFL-*qF | ATCGCACTACCGGTCACTCATT |
|  | *GmRFL-*qR | AAACTCCCGTGTCGGCTTG |
|  | *GmEIL1b-*qF | TGTGAGGCAGTCTAAGTGTCT |
|  | *GmEIL1b-*qR | GGGCAATAGTCAGGGTAAAG |
|  | *GmEIL1c-*qF | AGCCAACATGCCCAACAATC |
|  | *GmEIL1c-*qR | AAGGGGACCCAAACATCAAAT |
|  | *GmEIL1d-*qF | GATGAAGCTAACATGCCCAACA |
|  | *GmEIL1d-*qR | CCAAATCACAAAGGGAACCAA |
|  | *GmCTR1-*qF： | ATTGTTTTGTTTATGGGGGCA |
|  | *GmCTR1-*qR： | AAGCCATACCAAGCCTACGC |
|  | *GmETR1-*qF： | CAATGGGGTTAGCAGGACAGT |
|  | *GmETR1-*qR： | TCCAGAGAAACAACACGCAGA |
|  | *GmERS1-*qF： | TGGTGCCTTTATTGTTCTCTGTG |
|  | *GmERS1-*qR： | ACAAGCATCAGTGCCGTCG |
|  | *GmETR2-*qF： | CCTTGTAATCATGGTTTTCCTCA |
|  | *GmETR2-*qR： | CAGAGATGGCTATGGTAATGGAC |
|  | *GmERS2-*qF： | AACTACCATTACCCTCCTCACCT |
|  | *GmERS2-*qR： | TCCAACCTCCCTCCCAAGA |
|  | *GmEIN4-*qF： | TAAACAGGACCGTGACCAAGAA |
|  | *GmEIN4-*qR： | GCACTCAGGCACTCAAACCC |
|  | *GmEIN2-*qF： | TGTGTGTCTTCAGTGGCCTTTA |
|  | *GmEIN2-*qR： | CACCTGTTCCATTGGCGATA |
|  | *GmACS02-*qF： | TGTAAGCGTTGCCGAAGTGA |
|  | *GmACS02-*qR： | AACCCTGAAGCCAGGAAACC |
|  | *GmACS09-*qF： | CAGATGGGTCTTGCTGAAAACCAG |
|  | *GmACS09-*qR： | GAAATGGCAAATTTTATGTCAA |
|  | *GmACO3-*qF： | GTTAACCTTGGTGACCAAATTGA |
|  | *GmACO3-*qR： | TTCCCACCCCATTTTATCCCGG |
|  | *GmPR1a-*qF： | TGAAAATGTGGGTTGATGAGAAAT |
|  | *GmPR1a-*qR： | AAGTGATGAAAGTGCCTCCGTT |
| **GFP** | *GmEIL1-*GF | CAGATCT GATGATGATGTTTGAAGATA |
|  | *GmEIL1-*GR | CACTAGT CTGGTACCAAATTGAAAC |
